# Molecular signatures of aneuploidy-driven adaptive evolution

**DOI:** 10.1038/s41467-019-13669-2

**Published:** 2020-01-30

**Authors:** Alaattin Kaya, Marco Mariotti, Alexander Tyshkovskiy, Xuming Zhou, Michelle L. Hulke, Siming Ma, Maxim V. Gerashchenko, Amnon Koren, Vadim N. Gladyshev

**Affiliations:** 1 Division of Genetics, Department of Medicine, Brigham and Women’s Hospital and Harvard Medical School, Boston, MA 02115 USA; 2 Department of Biology, Virginia Commonwealth University, Richmond, VA 23284 USA; 3 Center for Data-Intensive Biomedicine and Biotechnology, Skolkovo Institute of Science and Technology, Moscow, 143028 Russia; 4 Belozersky Institute of Physico-Chemical Biology, Moscow State University, Moscow, 119234 Russia; 5 Cornell University Department of Molecular Biology and Genetics, 526 Campus Road, Ithaca, NY 14853 USA

**Keywords:** Saccharomyces cerevisiae, Evolutionary genetics, Experimental evolution, Aneuploidy

## Abstract

Alteration of normal ploidy (aneuploidy) can have a number of opposing effects, such as unbalancing protein abundances and inhibiting cell growth but also accelerating genetic diversification and rapid adaptation. The interplay of these detrimental and beneficial effects remains puzzling. Here, to understand how cells develop tolerance to aneuploidy, we subject disomic (i.e. with an extra chromosome copy) strains of yeast to long-term experimental evolution under strong selection, by forcing disomy maintenance and daily population dilution. We characterize mutations, karyotype alterations and gene expression changes, and dissect the associated molecular strategies. Cells with different extra chromosomes accumulated mutations at distinct rates and displayed diverse adaptive events. They tended to evolve towards normal ploidy through chromosomal DNA loss and gene expression changes. We identify genes with recurrent mutations and altered expression in multiple lines, revealing a variant that improves growth under genotoxic stresses. These findings support rapid evolvability of disomic strains that can be used to characterize fitness effects of mutations under different stress conditions.

## Abstract (web summary)

Aneuploidy (abnormal chromosome number) can enable rapid adaptation to stress conditions, but it also entails fitness costs from gene imbalance. Here, the authors experimentally evolve yeast while forcing maintenance of aneuploidy to identify the mechanisms that promote tolerance of aneuploidy.

## Introduction

Whole chromosome gains and losses lead to unbalanced karyotype, a phenomenon known as aneuploidy. Aneuploidy is frequently observed in eukaryotes and has been shown to affect cellular physiology in organisms from yeast to human^1^. The extra chromosomes delay cellular growth, interfere with embryonic development, increase genomic instability, and promote aging^1–5^. Impaired proliferation is a common feature of any type of karyotype alteration, including aneuploidy^2,6^. Having an extra chromosome has been shown to extend the G1 phase and decrease the growth rate in yeast^6^, partly due to gene expression imbalance^1,6^. However, studies in aneuploid yeast strains have shown that the slow growth rate of the initial clonal population can be restored within as few as 20 generations of adaptive evolution. The growth restoration was ascribed to a handful of mutations providing tolerance to aneuploidy^7^.

Many cancer cells are characterized by aneuploidy and rapid proliferation^8^, suggesting that aneuploidy can be advantageous for adaptation, if the initial challenge of gene imbalance can be overcome. Indeed, several studies indicated that aneuploidy allows cell populations to rapidly explore adaptive mechanisms by increasing the frequency of beneficial mutations and changing the mean fitness of the population^9–11^. Rapid aneuploidy-driven adaptation was observed for diverse genetic and environmental perturbations (e.g., telomerase deficiency^12^, heat shock^13^, oxidative stress and antifungal drug resistance^14–16^ and was attributed to copy number changes of specific genes on the duplicated chromosome. The cellular response to aneuploidy has been analyzed at the transcriptome level in plants^17^, mouse^18^ and human^19^ cell lines, and at both transcriptome and proteome levels in budding yeast^20,21^. These studies showed that aneuploidy can accelerate phenotypic variation and drive adaptation to stress. They also provided valuable insights on the mechanisms of cellular response to aneuploidy and adaptation to stress. However, much is left to learn about how cells can evolve to tolerate aneuploidy itself, allowing them to profit from rapid adaptation without succumbing to the liability of gene imbalance.

Disomic (i.e., possessing an extra copy of one or more chromosomes) yeast strains were shown to feature increased genomic instability, assessed by fluctuation assays using genetic markers, such as uracil (URA3) and canavanine (CAN1)^4^. These methods may be used to estimate mutation rates but have limitations in interpreting the diversity and specificity of mutant phenotypes when applied to whole genomes. In this study, to understand the mechanisms through which cells respond to aneuploidy and develop tolerance leading to fitness restoration, we subjected laboratory generated yeast haploid disomic strains^6,18^ with different extra chromosomes to long-term experimental evolution, aiming to uncover adaptive mechanisms of the response to aneuploidy stress. Our approach ensures that both phenotypic changes and their underlying genomic responses were triggered by the intrinsic chromosome copy number alteration during the experiment in a well-controlled environment. By applying genome and transcriptome sequencing, we uncover mutations, karyotype alterations, and gene expression changes that occurred in disomic strains during adaptive evolution. Our study illustrates a variety of mechanisms that support the response to aneuploidy. While aneuploid cells exhibit disome-specific mutational spectra, we also observe common trends across strains. Cells show a clear tendency towards the restoration of their euploid status, either by losing portions of the extra chromosome, or by attenuating the gene expression change induced by disomy. Specific pathways are found to be consistently altered throughout disomic strains, presumably as a response to aneuploidy. Among these pathways, we uncover adaptive molecular mechanisms that reduce the negative effects of gene and protein imbalance.

## Results

### Adaptive evolution leads to restoration of growth rates

**Fig. 1:**
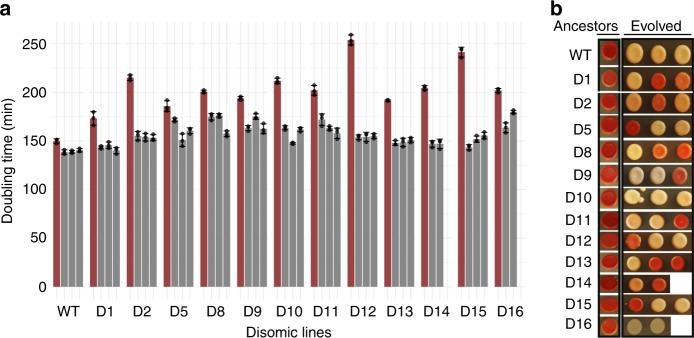
Phenotype changes in evolved aneuploid yeast lines. **a** Doubling time of ancestor and evolved lines. Cells were grown at 30 °C, and OD_600_ measurements were taken at 15 min intervals using a Biotek-Epoch2 instrument until the cells reached early stationary phase. Doubling time was calculated with a custom R script by analyzing fitting spline function from growth curve slopes. Bar graphs represent mean of three measurement and error bars are SEM. Red bars represent the doubling time of ancestor strains for both WT and disomes. Black bars show the doubling time of evolved lines for corresponding strains. Mean values are shown; error bars represent SD. Source data for doubling times is presented as source data file. **b** Color changes from red to white in evolved lines.

We carried out a laboratory evolution experiment with 12 disomic yeast strains (each with an extra copy of chromosomes I, II, V, VIII, IX, X, XI, XII, XIII, XIV, XV or XVI; hereinafter, referred to as “disome D1”, “disome D2”, etc.) (Supplementary Fig. 1). Duplications of chromosomes III, IV, VI, and VII led to lethality or very slow growth and therefore could not be analyzed in our study. By analyzing 105 clinical isolates and 1011 wild isolates of *Saccharomyces*^22,23^, we found that the lethal effect of chromosome III, IV, VI, and VII duplications is specific to our strain background (W303), since natural occurrences of whole or partial copy number alterations could be found for each of them among 386 aneuploid WT isolates (Supplementary Fig. 2). Consistent with previous data^2,6^, all twelve disomes we analyzed were characterized by slower growth compared to wild-type (euploid-WT) control cells (Fig. 1a). This also agrees with the observation that aneuploid wild isolates are characterized by reduced growth compared to their euploid counterparts^23^. Thus, the growth defect associated to aneuploidy is not artifactual, although we must note that W303 may be more sensitive to aneuploidy compared to wild isolates^24,25^.

Disome populations were cultivated in parallel under adaptive growth for 1,200 generations in selective medium in the absence of stress, with three replicates per strain. Strains were kept under continuous histidine (*HIS3*) and kanamycin (*KANMX*) selection to maintain disomy (each copy of the duplicated chromosome contained one selection marker). We then measured doubling time of ancestral and evolved disomes and WT cells to estimate growth rate. Consistent with previous reports^7,20^, in our hands all disomic lines improved growth rate after adaptive evolution (Fig. 1a). On average, the evolved WT populations decreased their doubling time by 8%, whereas the evolved disomic strains decreased it by 16–64%. Six disomic strains (D1, D2, D12, D13, D14, and D15) restored the doubling time to within ±10% of the doubling time of WT cells. The disomes with the most severe initial growth impairment (D2, D12 and D15) displayed the largest growth improvement (39%, 64% and 60%, respectively (Fig. 1a).

Notably, we observed distinct color changes, from red to pink or white, over the course of the experiment (Fig. 1b). Both disomic and WT strains were adenine auxotrophs due to non-functional *ADE1 or ADE2* genes (coding for N-succinyl-5-aminoimidazole-4-carboxamide ribotide synthase, phosphoribosylaminoimidazole carboxylase), so that all the ancestor strains formed red-colored colonies when plated on solid selective medium. The red pigment derived from the polymerization of ribosylaminoimidazole (AIR)^26,27^ in aerobically growing cells, which causes DNA cleavage^27^ and leads to growth defects^26^. It was reported that, in these conditions, spontaneous suppressor mutations may occur in genes upstream in the adenine pathway (*ADE4* through *ADE8*) to block AIR formation, and/or in genes involved in oxidative metabolism to prevent oxidation of AIR, resulting in white colored mutants that grow faster than their red relatives^27^. Indeed, at the end of our long-term adaptive growth, all three evolved WT lines formed cream-colored smooth colonies (Fig. 1b) and concomitantly decreased doubling time by 7.7%, supporting the idea that mutations in the adenine pathway contributed to their growth improvement. However, while many evolved disomic strains also appeared white, their improvement in growth was much larger and could not be explained by this mutation alone. In addition, many evolved disomic lines remained red but still grew much faster than their ancestral strains, indicating that aneuploidy, rather than adenine deficiency, dominated the evolutionary trajectories of disomic lines.

### Karyotype dynamics in evolved disomic populations

**Fig. 2:**
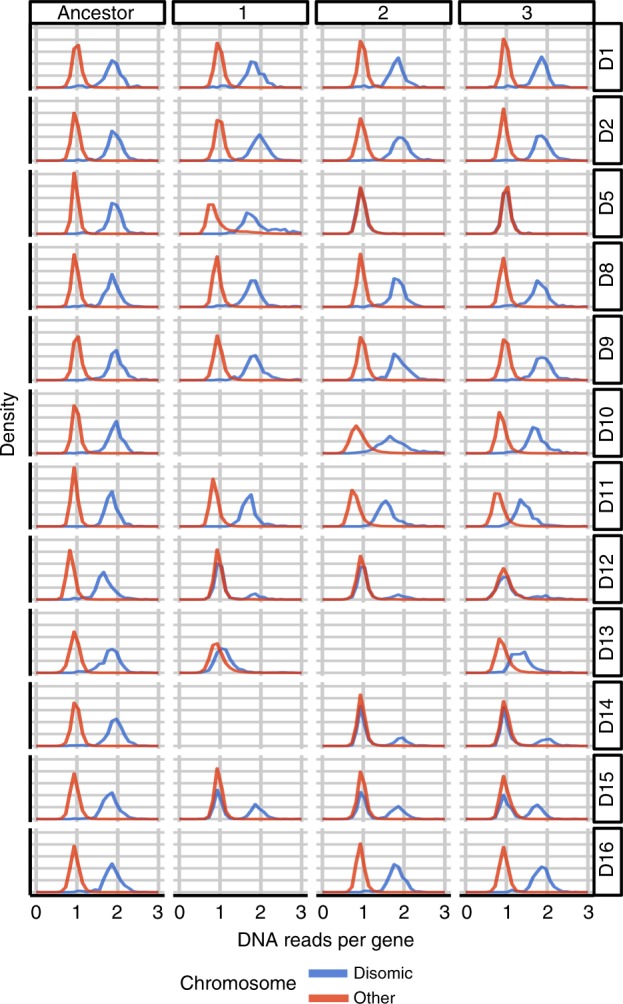
Karyotype of evolved disomic lines. The plot shows the distribution of genomic read density per gene. Values are normalized per sample by the total number of mapped reads, and per gene to the WT value. Genes were split in two groups, separating those located on the disomic chromosome (chromosome I for D1, chromosome II for D2, etc.; shown in blue) from the rest (red). This representation allows to readily identify losses of the extra chromosome (e.g. complete losses in D5 lines 2 and 3). A more detailed read density heat map is shown in Supplementary Fig. 3.

**Fig. 3:**
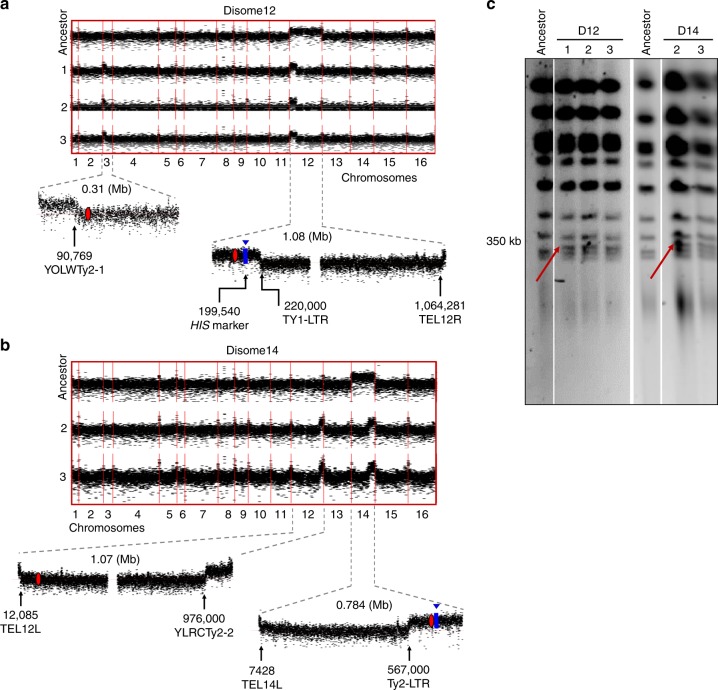
Karyotype of evolved disome D12 and D14 lines. **a** Karyotype of ancestor and evolved disome D12 lines and **b** disome D14 lines. Read depth was calculated in 100-bp windows. The lower sections of these panels zoom in the chromosomal regions with duplication break points. Location of selection markers is shown in blue, and centromeres in red. Gaps in chromosome XII correspond to rDNA regions that were removed for visualization purposes. **c** Pulse-field gel analysis showing the distribution of chromosome sizes in ancestor and evolved lines of disomes D12 and D14. Red arrows indicate the appearance of new chromosomes in both disomes. Source data for uncut gels is provided as a source data file.

We hypothesized that the restoration of growth of evolved disomic lines was associated with compensatory mechanisms in response to chromosomal imbalance. To elucidate them, we performed whole genome sequencing of 35 evolved lines (32 disomic, 3 WT lines) and their respective ancestors. We then characterized the karyotype changes in every line, by analyzing genome-wide read coverage (Supplementary Fig. 3). Previous studies highlighted various features of genome instability in aneuploid cells: the karyotype of aneuploid cells often changes during long-term growth^22^, and aneuploid strains are prone to changes in chromosome number even during short-term heat stress^28^. Notably, frequent karyotypic changes were observed both in wild and lab aneuploid strains^28,29^. Our analysis showed that 22 out of 32 evolved disomic lines retained the karyotype of their ancestral strains, whereas 10 lines representing four disomes (D5, D12, D14, and D15) acquired partial or whole chromosome losses and/or large segmental amplifications (Fig. 2, Supplementary Fig. 3). Two of the three evolved lines of disome D5 lost the extra chromosome completely (Supplementary Fig. 4), while all the lines of disomes D15 lost the duplicated chromosome partially. Interestingly, all disome D12 and D14 lines lost a part of the duplicated chromosome but acquired large segmental duplications of other chromosomes (Fig. 3).

The complete loss of the duplicated chromosome in disome D5 lines was surprising, since each chromosome copy carried a different selection marker (HIS or KANMX at the same genomic location). However, we observed many genomic reads mapped to *HIS3* and *KANMX*, indicating that the markers were still present in these cells. We then de novo assembled chromosome V and found both markers on the same chromosome copy. In this strain, selection markers were located very close to the telomeric region of the right arm, where long stretches of repetitive elements and transposons are located (ChrV:31694-33207). This may have facilitated unequal homologous recombination of selection markers, resulting in their co-occurrence on the same chromosome V molecule, and thus allowing subsequent restoration of normal ploidy through the loss of the other copy (Supplementary Fig. 4).

Another karyotype change was observed in disomes D12 and D14. Here, only a portion of the original duplicated chromosome remained, along with newly emerged segmental duplications of chromosome III and chromosome XII, respectively (Fig. 3a, b). Notably, the breakpoints of these segments were all located at the loci of transposable elements and telomeric regions. In the evolved D12 lines, the retained portion of the originally duplicated chromosome contained the centromere, selection marker, and telomeric regions. The other amplified chromosome segment (III) did not include the centromere (Fig. 3a). The transposable elements located at breakpoints showed high sequence similarity, suggesting that homologous recombination occurred between the two amplified segments. The same pattern was observed in the evolved disome D14 lines: the remaining segment of the amplified chromosome XIV contained the centromere as well as the selection marker, while the newly amplified part of chromosome XII had breakpoints at highly similar transposable elements (Fig. 3b). Based on these observations, we hypothesized that additional small chromosomes might have formed from the fusion of segments in two chromosomes, including centromere and telomeric regions. Based on the breakpoint locations, we estimated the size of these predicted newly formed chromosomes to be ~350 kb, both for D12 and D14. Using pulse-field gel electrophoresis (PFGE), we analyzed the chromosome size profiles of these evolved lines, and indeed detected novel bands of the expected size in each strain (Fig. 3c). Since the newly formed chromosomes contained the centromere, they probably segregated during mitosis and remained stable throughout subsequent generations in these lines. These observations support the idea that aneuploidy is characterized by high genomic instability, as it triggered diverse karyotype alterations across strains. Since some occurred recurrently in several evolved lines of the same strains, it is likely that these alterations represent adaptive responses. We suppose that aneuploidy may have enhanced the occurrence of structural variants; however, due to the sequencing protocol employed (short reads), we could not investigate these in detail.

### Spectra of mutations fixed in disomic strains

**Fig. 4:**
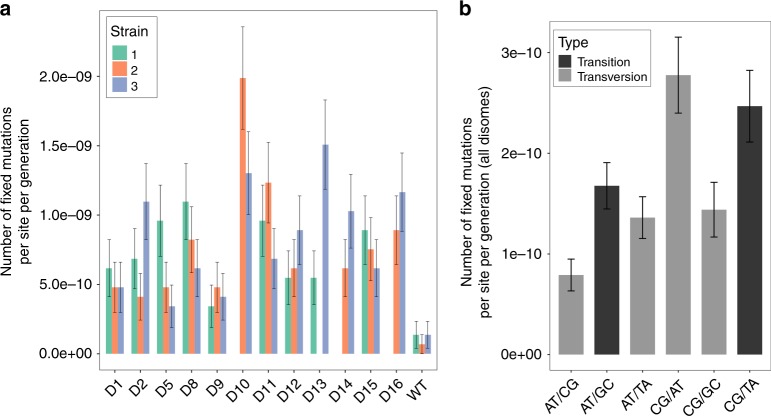
De novo mutations during experimental evolution. **a** Number of de novo mutations per site per generation fixed during experimental evolution of disomes and WT. **b** Apparent mutation rates split by mutation type. Error bars represents standard errors computed as in ref. ^74^. Source data for the number of mutations and the rate are provided as a source data file.

In addition to karyotype changes, we analyzed de novo mutations accumulated over the course of laboratory evolution in both disomic and WT lines. To do this, we compared the genomes of evolved lines to their corresponding ancestral strains and focused on the variants absent in the ancestral genome. This analysis revealed 253 de novo nucleotide substitutions and 120 small insertions/deletions (indels) across the 32 evolved disomic lines (Table 1, Supplementary Data 1), with an average of 7.9 base substitutions and 3.75 small indels per evolved line. D10 was the most mutated disome (averaging 14.5 substitutions and 9.5 indels per evolved line), while D9 was the least mutated (averaging 4.3 substitutions and 1.6 indels). Overall, we observed that the average number of mutations did not significantly correlate with the length of the duplicated chromosome (Pearson correlation = 0.26, *p* = 0.41). We computed the “apparent mutation rate” of each evolved line by dividing the number of observed de novo mutations by genome size and by the number of generations. This rate differs from the true spontaneous mutation rate, estimated in mutation-accumulation experiments with frequent bottlenecks^30^, in that here selective forces can alter the dynamics of variant fixation. WT cells accumulated 1.67 substitutions and indels per evolved strain, implying a rate of fixed mutations, 1.1 × 10^−10^ per nucleotide per generation, slightly lower than the previously reported spontaneous mutation rate of ~3 × 10^−10^^30^. The apparent mutation rate in disomic strains was higher than in WT, averaging 8.4 × 10^−10^ and ranging from 4.1 × 10^−10^ (disome D9) to 16.5 × 10^−10^ (disome D10) (Fig. 4a). The higher rate in disomic lines may be explained either by an increase in spontaneous mutation rate, or by an increase in positive selection, promoting selective sweeps of variants beneficial in disomic background (or by a combination of both). Our design does not allow to differentiate these two effects. Yet, we observed that the apparent mutation rate in our disomic lines was higher than WT for both synonymous (average 5.5 × 10^−10^ vs 1.4 × 10^−10^) and non-synonymous (average 3.5 × 10^−10^ vs 1.3 × 10^−10^) substitutions. Although hitchhiking in selective sweeps may also be inflating the apparent rate, our observations are consistent with the disomic strains having increased spontaneous mutation rates, as shown in other studies^4,31^. We observed bias in the localization of mutations towards noncoding regions (Table 1), significantly deviating from uniform distribution (~74% of the yeast genome consists of coding sequences^32^). The effect was statistically significant for both substitutions and indels, and was stronger for indels (binomial test, *p*_sub_ = 6.9 × 10^−12^ and *p*_ind_ < 2.2 × 10^−12^, respectively). These observations are consistent with purifying selection acting on coding sequences. Notably, we found that mutations in disomic lines were preferentially located on the duplicated chromosomes (binomial test, adjusted *p* < 0.05 for all disomes except D11; see “Methods”). The significance of this pattern is discussed later.

**Table 1:** Mutations observed in evolved lines compared to their ancestors.

Substitution/Strain	WT	D1	D2	D5	D8	D9	D10	D11	D12	D13	D14	D15	D16
Chromosome length (kb)		230	813	576	562	439	745	666	1080	924	780	1090	948
Total number of mutations (substitutions and indels)	5	23	32	26	37	18	48	42	30	30	24	33	30
Mutations on duplicated chr.		7	8	5	9	5	14	7	11	9	7	12	11
Total substitutions (cod/ncod)	3/1	5/11	3/11	15/6	15/15	8/5	19/10	13/10	13/8	9/7	8/14	13/13	13/9
Missense	2	3	2	10	10	6	11	8	9	6	6	10	8
Nonsense	0	1	0	0	1	1	1	0	0	0	0	1	2
Silent	1	1	1	5	4	1	7	5	4	3	2	2	3
Total indels (cod/ncod)	1/0	2/5	3/15	0/5	1/6	0/5	13/6	5/14	2/7	2/12	0/2	1/6	2/6

Total number of mutations across evolved lines of the same ancestors are shown

In our study, the rate of G:C → A:T transitions did not exceed the A:T → G:C rate, unlike some previous WT yeast studies^33,34^. However, C:G → A:T transversion was the most prominent transversion mutation (Fig. 4b), in agreement with earlier reports^34^. These results may indicate that oxidative conversion of guanine to 7,8-dihydro-8-oxoguanine (8-oxo-G)^35^, rather than spontaneous deamination of methylated cytosines, is the major driver of mutations in disomic strains, consistent with the observation that many disomic strains are characterized by increased levels of reactive oxygen species^6,20^. Moreover, we examined variants across the entire genome to identify potential positional effects and to determine if any of these disomic cells were affected by DNA replication stress. At the global level (i.e., when mutations were combined for all evolved lines), there was no association between the position of mutations and the known replication timing of genomic regions. This observation suggests that none of these chromosome duplications altered DNA replication timing at the onset of S phase, which is also consistent with disomic strains having extended G1 phase but not S phase (Supplementary Fig. 5a, b)^6^. Finally, we compared the pattern of mutations fixed in disomic lines with mutational signatures of human cancer (COSMIC signatures)^36^. Interestingly, the disomic yeast mutations resembled COSMIC signature 5 (Supplementary Fig. 6a, b). This signature is being reported as found in all cancer types with the number of mutations in most cancer samples and normal cells correlating with the age of the individual. While the significance of this signature is yet unclear, our observation may suggest a link with aneuploidy-related stress, consistent with ~90% of cancers of any type having detectable aneuploidy^8^. In addition, an age-associated increase in aneuploidy was shown previously^37^, suggesting that an age-associated increase in karyotype changes might also contribute to the mutational trajectories observed during aging. Taken together, these data indicate that aneuploid genomes are characterized by increased genomic instability, though its degree varies substantially across different disomes.

### Recurrent mutations during the evolution of disomic lines

To gain insights into the molecular effects of mutations on aneuploidy evolution, we analyzed the genes commonly mutated in evolved lines. There were many spontaneous suppressor mutations in genes in the adenine pathway (*ADE4* through *ADE8*) to block AIR formation, consistent with the color changes in the evolved lines (Fig. 1b). However, several strains showed white phenotype but had no mutations in the adenine pathway, so we examined if they were respiration deficient (which would also result in white phenotype^27^). For this, we analyzed mitochondrial DNA (mtDNA) copy number and performed growth assays in the medium containing glycerol as a carbon source. We found that many evolved lines increased mtDNA quantity compared to their ancestral strains (Supplementary Fig. 7a), as was previously shown for aneuploid human and mouse embryo^38,39^. All evolved lines of disomes D10, D11 and D13 increased mtDNA copy number up to fourfold, suggesting that this phenomenon may be triggered by the gene content of those duplicated chromosomes. All evolved lines could utilize glycerol as a carbon source, indicating that none of the disomic strains were respiratory deficient (Supplementary Fig. 7b). On the other hand, we observed recurrent mutations in the genes of the *SUP* family (*SUP3, SUP4, SUP5*), known as nonsense suppressors^40^. These genes normally encode for tyrosine tRNAs, and specific mutations in the wobble position of its anticodon allow them to read stop codons and suppress nonsense mutations^41^. Indeed, we found that all evolved white colonies without compensatory mutations in the adenine pathway carried the same C to A mutation in the wobble position of tyrosine tRNA (lines 1 and 2 of D11 and line 2 of D16; Supplementary Fig. 7c). As the result, the full-length *ADE2* could be produced by stop codon readthrough, ultimately resulting in the restoration of the adenine pathway and a change of colony color from red to white (Fig. 1).

**Fig. 5:**
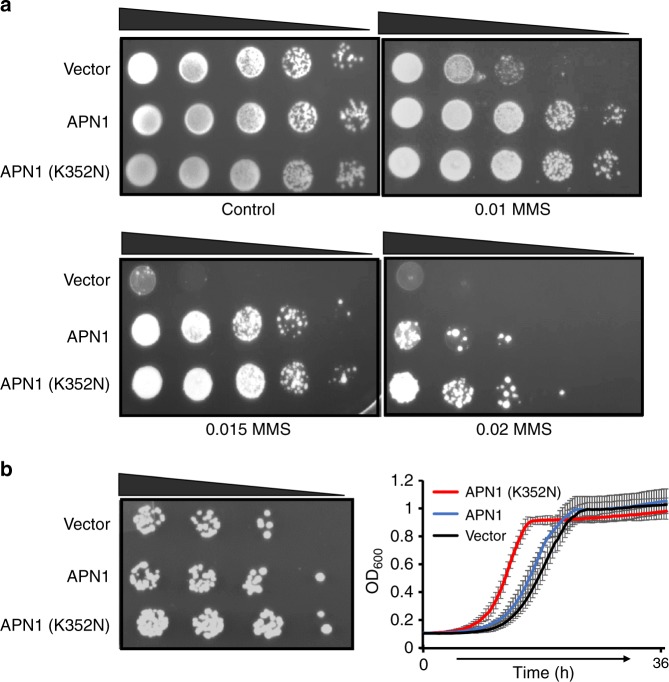
Effect of *APN1* K352N mutation on cell survival under genotoxic stress. **a** Spot assay for survival effect of *APN1* K352N mutation under MMS stress. Plates with YNB medium without His were supplemented with indicated amounts of MMS (%), and *ΔAPN1* cells harboring an empty vector (p413ADH), or vectors expressing endogenous *APN1* or mutated *APN1* spotted with 10-fold dilution onto plates. **b** Spot (left) and growth (right) assays for assessing viability of cells with mutated *APN1* in response to oxidative DNA damage using a thiol peroxidase null strain (Δ7). Each spot assay was repeated three times, and the growth curve shown is the mean of three independent measurements. Expression of *APN1* K352N mutation significantly improved the growth of Δ7 cells, compared to cells expressing non-mutated *APN1* (two-sided *t* test, *p* ≤ 0.001).

Our analysis also identified another gene, *APN1*, which was recurrently mutated across all evolved lines of disome D12. *APN1* encodes a major apurinic/apyrimidinic endonuclease involved in oxidative and alkylation DNA damage repair^42^. All evolved disome D12 lines harbored the same nucleotide change (T → G) that replaced Lys352 with Asn in Apn1 (K352N variant). Since it is highly improbable that the same mutation occurred de novo in all evolved lines, we hypothesized that the variant was already present in the ancestral strain population and was later driven to fixation by positive selection during adaptive growth. Indeed, we confirmed through Sanger sequencing the existence of this variant at low frequency in the ancestral disome population (Supplementary Fig. 8). Apn1 functions in the repair of oxidative and alkylation DNA damage^42^ and prevents the accumulation of repair-associated DNA breaks in yeast^43^. We tested the possibility that the K352N mutation enhanced protective activity and was thus selected for its beneficial effect on the proliferation of disomic cells, known to suffer from intrinsic oxidative stress^6,20^. Oxidative DNA damage marker 8-oxo-G was the most common base modification among our disomic strains including D12. Even though the canonical pathway of mismatch repair appears to be typically associated with repair of 8-oxo-G lesions, the 3′ → 5′ exonuclease activity of Apn1 was found to support an alternative pathway for repairing this form of damage^44^. We decided to dissect the fitness effect of this single base pair substitution in response to the DNA alkylating agent, methyl methanesulfonate (MMS), and under conditions of oxidative DNA damage. First, we introduced constructs harboring mutated (K352N) and non-mutated *APN1* in *APN1*-knockout (*ΔAPN1*) euploid cells and tested their ability to grow on the MMS-containing medium. We observed that the mutated *APN1* conferred improved viability against MMS genotoxicity (Fig. 5a). Second, we tested the fitness effect of this substitution in the thiol peroxidase null yeast (Δ7), a strain featuring a high mutator phenotype due to the absence of eight major oxidoreductases^45^. We observed that expression of the mutated version of *APN1* increased viability of Δ7 cells (Fig. 5b). These data suggest that this single base substitution in *APN1* provides a mild fitness benefit under both alkylation and oxidative stresses. We further extended this finding by analyzing the effect of *APN1* K352N on growth of all 12 disomic lines under MMS stress. We thus introduced constructs harboring mutated (K352N) and non-mutated *APN1* in disomic and WT cells and tested for their ability to grow on the MMS-containing medium. We observed that the mutated *APN1* improved viability for all disomic lines except D11, D15 and D16 (Supplementary Fig. 9a, b). This finding supports a protective effect of *APN1* K352N in stress conditions, consistent with the reported intrinsic oxidative stress in disomic strains^20^. To understand the mechanism by which K352N improved viability under stress, we modeled Apn1 structure (Supplementary Fig. 10). The residue 352 was located at the end of the double helix tail of Apn1, whose functional properties are unknown. We speculate that this mutation may alter protein-protein interactions or DNA binding properties of Apn1.

We further observed that some genes accumulated distinct coding sequence mutations in multiple evolved disomic strains (Supplementary Data 2). Recurrent mutations of the same genes likely represent adaptation to aneuploidy. These genes include ubiquitin (*UBI4*); a protein kinase that is part of the growth control pathway (*SCH9*); essential components of the RSC chromatin remodeling complex and condensin complex proteins (*RSC8* and *SMC4*); a putative protein of unknown function with similarity to sialidase (*YMR317W*); and a mitochondrial protein with unknown function (*FMP27*). Although mapping to the same genes, these mutations were at different loci in different disomic strains, indicating that they emerged independently in the various lines.

**Fig. 6:**
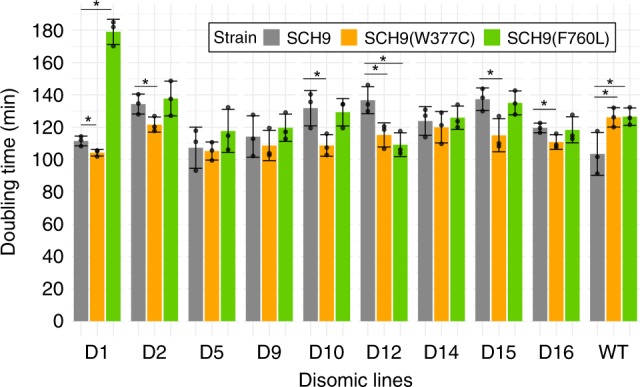
Effect of *SCH9* W377C and F760L mutations on cell growth. The effect of the *SCH9*-W377C and *SCH9*-F760L alleles on cell growth was analyzed with OD_600_ measurement in triplicates. Disomic and WT strains were transformed with a linear PCR product of *SCH9* (control, gray bars) or one of the mutated alleles (orange and green bars), fused with *natMX6* marker (see Methods). Overnight cultures were diluted to 0.01 and growth was monitored for 2 days with BioTech Epoch2 plate reader. Error bars represent standard deviations. P-values for each condition were computed with two-sided t-test between each mutated allele and control and are supplied in Supplementary Data 3. Significant changes are marked with an asterisk (*). Source data for doubling times is provided in Supplementary Data 3.

We also considered mutations in intergenic regions, which we assigned to gene promoters based on their distances to transcription start sites (see “Methods”) (Supplementary Fig. 11). We identified 84 base substitutions and 67 indels in the promoter sites of 135 genes (Supplementary Data 1). The promoters of six genes were commonly mutated in different disomic strains (Supplementary Data 2): a transcriptional regulator (*ERT1*); an essential nuclear protein required for maturation of 25S and 5.8S rRNAs (*MAK16*); a protein involved in ribosome biogenesis (*SDA1*); a histone-fold protein (*MHF2*); and, once again, the *SCH9* kinase. Among them, the *SCH9* gene presented both promoter mutations in evolved D2 and D15 lines, and coding region mutations in evolved D10 and D13 lines. *SCH9* is the ortholog of mammalian S6 kinase (required for TOR1-mediated regulation of ribosome biogenesis and translation) and it was previously found to stabilize the tetraploid phenotype in yeast cells^46^. We hypothesized that *SCH9* acted in the translational regulation of disomic strains to decrease the proteotoxic effects of duplicated chromosomes, and decided to test the effect of mutations observed in evolved disomes on proliferation. We focused on coding sequence mutations in *SCH9*, W377C and F760L, which were found in D10 line 3 and D13 line 1, respectively. We employed fragments harboring either non-mutated or mutated *SCH9* to replace the endogenous copy in the genome of disomic strains and monitored their growth. We could assay 9 disomic strains, in which we successfully replaced the endogenous copy with the mutated *SCH9*. While expression of both mutant versions of *SCH9* caused a significant growth delay in WT cells, the response of disomic lines was diverse (Fig. 6). In six disomic strains, *SCH9* W377C conferred significantly improved growth rate compared to non-mutated *SCH9*, indicating that this mutation may contribute to improve proliferation under aneuploidy stress (Fig. 6, Supplementary Data 3). *SCH9* F760L, in contrast, did not alter the doubling time in the great majority of disomic strains. It may be that this mutation provides fitness benefit only in the genetic background where it arose (D13, which unfortunately we could not assay here); or it may effectively be a neutral variant, located on *SCH9* simply by chance (which we deem highly unlikely).

In addition to *SCH9*, the occurrence of recurrent mutations in ribosome associated proteins *SDA1* and *MAK16* further reinforces the idea that regulation of translation is a key feature of the evolutionary response to aneuploidy. Furthermore, recurrent mutations in RSC complex (*RSC8* and *SMC4*) and histone-fold (*MHF2*) proteins may affect chromatin condensation and, ultimately, gene expression regulation in disomic strains. Our results support the idea that mutations recurrently fixed in the same genes represent adaptive responses in transcriptional and translational regulation to gene imbalance caused by aneuploidy and point to specific pathways as potential drivers of genetic adaptation to aneuploidy stress. Our study also shows that aneuploid yeast can be used for future studies to capture adaptive mutations under different stress conditions, since rapid evolvability drives adaptation with strong positive selection in these strains.

### Gene expression changes mark adaptation to aneuploidy

**Fig. 7:**
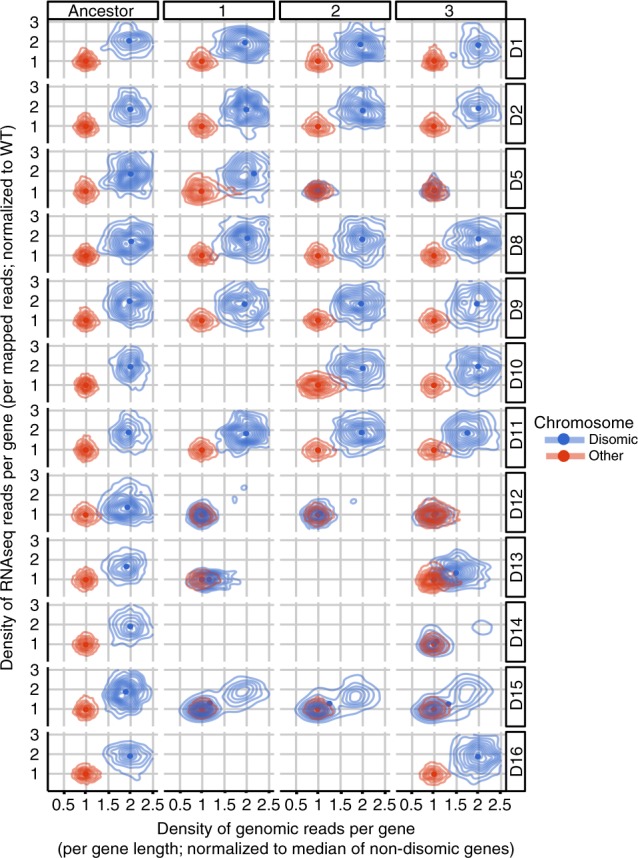
Relationship between gene expression and gene copy number in disomic lines. *X* axis represents gene copy number, estimated by the density of genomic reads; *Y* axis shows the level of gene expression, estimated by the density of RNAseq reads. Contour lines are used to display the distribution of all genes in the genome, split in two groups: those located on the disomic chromosome (e.g., chromosome II for D2; shown in blue) and the rest (red). Median expression values of each group are indicated with blue or red solid dots. The panels corresponding to samples with missing genomic and/or RNAseq data were left blank.

To investigate the transcriptional landscape of aneuploid strains throughout adaptive evolution, we obtained RNAseq profiles of all lines (evolved, ancestor disomic and WT strains). We then compared ancestor disomic lines with the WT line to outline the effect of aneuploidy on transcription. As expected, each ancestor disomic strain showed a dramatic increase in the expression of genes on the duplicated chromosome, with a clear karyotype-specific pattern of gene expression (Fig. 7, Supplementary Data 4). The normalized median expression of genes on disomic chromosomes varied across the ancestor disomic strains between 2.02 and 1.37-fold over WT with the mean of 1.8-fold (stdev = 0.17) (Fig. 7), consistent with previous studies^20^. We further compared the transcriptional profiles of evolved lines with their corresponding ancestral strains, reflecting the changes that occurred during evolution of aneuploid strains. Even after adaptive evolution, many evolved lines maintained a pattern of chromosome-specific elevated gene expression (Fig. 7)^7,20^. We also observed lineage-specific attenuation of gene expression in the strains that lost entire or large portions of the duplicated chromosomes, in agreement with the principle that gene expression level primarily depends on gene copy number. We then investigated whether evolved disomic strains exhibited any common expression changes during adaptive evolution, potentially related to regulatory mechanisms of tolerance. To address this question, we searched for genes commonly upregulated or downregulated across evolved lines. Our analysis revealed 274 such genes at FDR of 10% (see Methods). These genes segregated into functional categories (Supplementary Fig. 12a, Supplementary Data 5). 85 genes were upregulated and enriched in the GO term for ribosome biogenesis (adjusted *p* = 1.03 × 10^−17^), and 189 genes were downregulated and enriched for the amino acid synthetic pathway (adjusted *p* = 1.57 × 10^−18^), endonuclease activity (adjusted *p* = 4.85 × 10^−30^) and endopeptidase activity (adjusted *p* = 3.83 × 10^−19^) (Supplementary Fig. 12b).

**Fig. 8:**
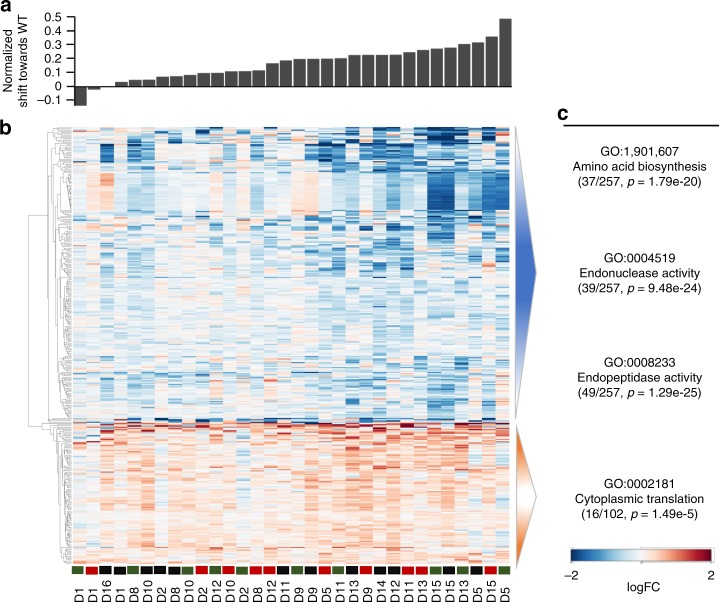
Changes of gene expression pattern associated with attenuation towards WT. **a** Evolved disomic strains, sorted by the normalized expression shift towards WT (see “Methods”). **b** Heat map of 359 genes whose expression correlates with the shift across evolved strains. Evolved lines of each disomic strains are shown in green (evolved line 1), red (evolved line 2) and black (evolved line 3). The list of genes with log2-fold changes along with adjusted *p*-values (*t*-test) can be found in Supplementary Data 5. **c** Enriched GO terms for genes. In parenthesis, the first number corresponds to the total number of genes in this GO category, and the second number indicates the total number of up-regulated or down-regulated genes. Enrichment adjusted *p*-values for each GO category are also shown.

To understand how evolved disomic strains changed their transcriptomes compared to the initial disomes, we further formulated a distance metric for RNA expression, which included genes with altered expression in ancestral disomes (see “Methods”). We used this metric to compare evolved disomic strains with WT, normalizing by the initial distance of ancestor disomes with WT. We observed that the great majority of evolved strains (30/32, 93%) decreased their distance to WT compared with their ancestors (Supplementary Fig. 13a). However, when comparing all samples using genome-wide expression profiles instead, evolved lines tended to cluster together with their ancestor disomes (Supplementary Fig. 13b). Therefore, while evolved disomic lines mostly retained transcriptional profiles of their ancestors, adaptive evolution specifically attenuated the largest expression alterations that were introduced by aneuploidy. We thus reformulated our distance metric in terms of the expression shift towards WT (equal to 1 min the distance as previously defined) (Fig. 8a). Attempting to elucidate common mechanisms of attenuation, we searched for genes whose expression correlated with such expression shift across evolved lines (Fig. 8b). At FDR of 10%, our analysis yielded 359 genes (Supplementary Data 5). 102 genes positively correlated with the shift towards WT, and were enriched for the GO term of cytoplasmic translation (adjusted *p* = 1.49e-5); the remaining 257 negatively correlated with the shift towards WT, and were enriched for amino acid biosynthesis (adjusted *p* = 1.79e-20), endonuclease activity (adjusted *p* = 9.48e-24) and endopeptidase activity (adjusted *p* = 1.29e-25) (Fig. 8c). We noticed that the set of shift-associated genes had a significant overlap with the set of genes differentially expressed across all evolved lines (Fisher exact test, *p* < 1e-30) (Supplementary Fig. 14).

**Fig. 9:**
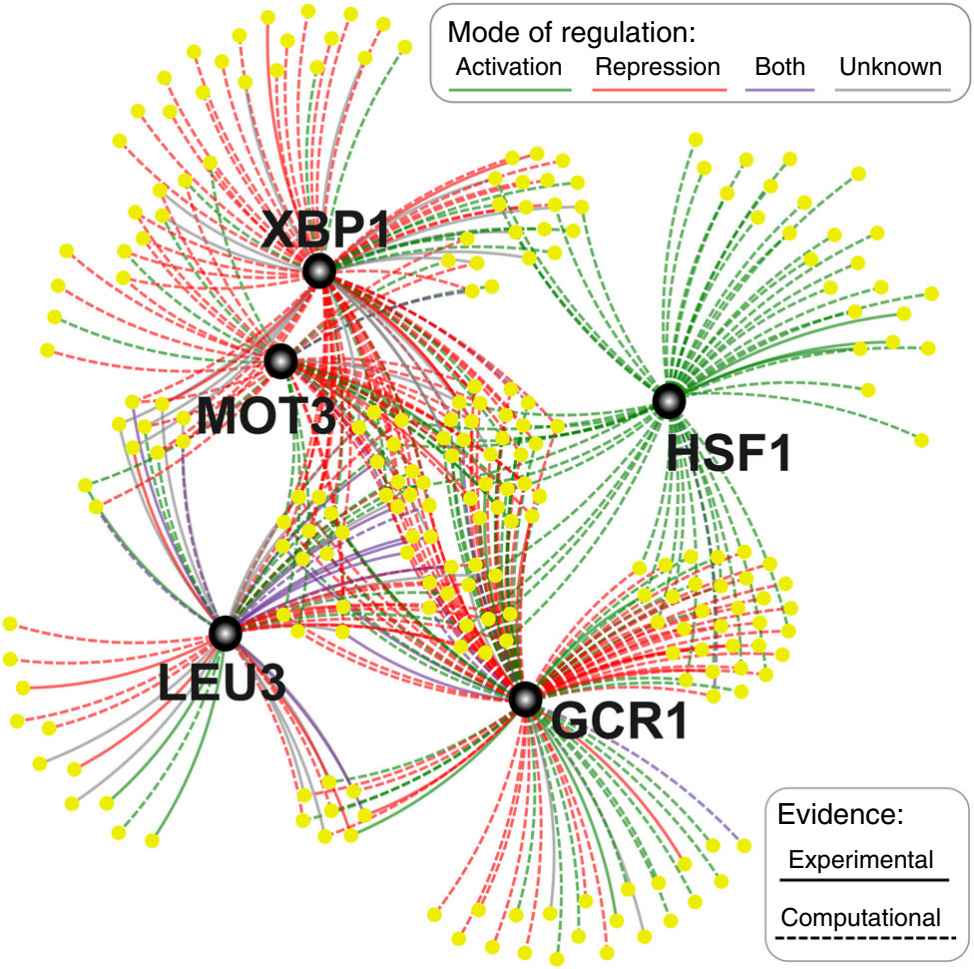
Transcriptional network of genes involved in gene expression shift towards WT. Black nodes indicate TFs whose motifs were enriched in the promoters of shift-associated genes (yellow nodes).

Finally, we searched for transcription factor (TF) binding motifs enriched in the promoter sequences of the genes associated with expression shift, aiming to identify TFs involved in the response to aneuploidy. Our analysis revealed significant enrichment of 5 TFs: *LEU3* (adjusted *p* = 7.9e-03), involved in branched chain amino acid biosynthesis; *MOT3* (adjusted *p* = 9.9e-04), regulates wide range of genes including glucose transporters; *GCR1* (adjusted *p* = 4.3e-09), regulating the expression of glycolytic and ribosomal genes; *XBP1* (adjusted *p* = 4.0e-09), regulating the expression of stress or starvation induced genes; and *HSF1* (adjusted *p* = 5.3e-04), activating multiple genes in response to stresses that include hyperthermia. Many of the shift-associated genes possessed motifs for more than one of these TFs (Fig. 9). We further explored the potential function of these TFs in response aneuploidy. First, we checked the mRNA expression of TFs in our evolved disomic lines. Notably, we found that the expression level change (evolved vs ancestral disome, fold change) significantly correlated with the expression shift towards WT for two TFs, *GCR1* (negative correlation, adjusted *p*-value = 0.015) and *XBP1* (positive correlation, adjusted *p*-value = 0.020) (Supplementary Fig. 15a). Next, we experimentally analyzed the effect on growth of TF deletion or over-expression across disomic lines. First, using a previously published data set^47^, we analyzed individual knockouts of three of these TFs (*GCR1* and *HSF1* are essential and cannot be deleted). Strikingly, our analysis revealed that deletion of *LEU3* significantly increased growth rate in six out of seven disomic strains, while *XBP1* or *MOT3* deletion had no significant effect (Supplementary Fig. 15b). Second, to assay the effect of increased TF expression, we prepared five constructs harboring the coding region of each TF along with 1 kb upstream, presumably resulting in native regulation with double expression. We transfected nine disomic lines, as well as WT cells as control, with these constructs individually. Our analysis showed that the growth of most disomic strains was unchanged upon increased expression of any of the analyzed TFs (Supplementary Fig. 15c, Supplementary Data 6). This may reflect the challenges of altering TF abundance by introducing an extra gene copy, as TF activity is often modulated by complex post-translational control involving protein localization, phosphorylation, ligand binding, or other biochemical modifications^48^. Our results suggest that altered activity of these TFs (particularly *LEU3*, *XBP1* and *GCR1*) may be relevant to mitigate the burden of aneuploidy on gene expression. Still, further investigation is required to address their direct role, and their mechanism of altered regulation. Altogether, our gene expression analysis revealed common regulatory mechanisms, including changes in protein translation, amino acid biosynthesis, and stress response, as potential key factors in the adaptation to aneuploidy.

## Discussion

In this study, we subjected lines of disomic yeast to long-term adaptive evolution to reveal mechanisms of tolerance to various chromosomal aneuploidies. In our evolution experiment, we chose a strategy of progressive dilution, rather than single colony bottleneck, to maximize the cumulative supply of beneficial mutations. Using selective markers to force the maintenance of aneuploidy, we were able to investigate the patterns of fixed mutations, karyotype alterations, and gene expression changes. This experimental strategy allowed us to extensively characterize responses to aneuploidy, uncovering several associated molecular mechanisms. Our analyses recapitulated well-known characteristics of aneuploidy and provided many new insights. Disomic cells featured increased genomic instability, i.e., evolved disomic lines sustained diverse karyotype alterations, and concomitantly presented an apparent mutation rate on average 10-fold higher than that of WT cells. The gene expression profiles of aneuploid cells showed marked differences with normal cells: genes on the duplicated chromosomes increased their expression levels 1.8-fold on average. The doubling time of ancestor disomic lines was clearly deteriorated compared to euploid cells.

However, adaptive evolution over ~1,200 generations partially restored the growth rate, sometimes approaching that of control cells (Fig. 1a). We then dissected the genetic mechanisms of such fitness restoration. Some phenotypic changes were not linked to aneuploidy and instead were related to our experimental setup. All strains in this study had a dysfunctional adenine pathway, and since this caused the accumulation of a deleterious red-pigmented AIR polymer, many strains (including WT) spontaneously fixed nonsense mutations to prevent AIR formation. Other cells restored the functional adenine pathway by mutating a Tyr-tRNA so that it recognized stop codons, suppressing the effect of the existing nonsense mutations.

Our analyses identified diverse mechanisms related to aneuploidy response. The most evident events involved large chromosomal rearrangements. Some strains (lines 2 and 3 of D5) managed to practically bypass our selection scheme to force continuous disomy, and reverted to the euploid status. Facilitated by the proximity of highly repetitive sequences, unequal homologous recombination brought the selection markers to the same chromosome molecule, subsequently allowing the loss of the extra copy. Similar events allowed other disomic lines to lose large portions of duplicated chromosomes, while retaining selection makers (D12, D14, D15), in some cases concomitantly with the formation of novel small chromosome bodies (D12, D14).

Disomic lines accumulated a number of mutations in coding and non-coding sequences during evolution. Notably, we found that these variants were preferentially located on the chromosome with an extra copy. This observation may be explained by either relaxed purifying selection on genes with a redundant copy, or by increased positive selection for variants compensating the imbalance in gene dosage. In the latter scenario, we would expect mutations to be fixed preferentially in the genes with the most extreme expression imbalance in disomic strains. Yet, mutated genes are indistinguishable from non-mutated in their distribution of expression fold change, both when we compare ancestral disomes with WT, or evolved lines with WT (Supplementary Fig. 16; all *p*-values two-sided *t*-tests > 0.6). Based on this, we favor relaxed purifying selection as most likely explanation for the biased localization of mutations on disomic chromosomes.

Next, we focused on genes that were recurrently, yet independently, mutated in multiple disomic strains, as an indication of positive selection. The known functions of these genes (Supplementary Data 2) are consistent with an adaptive role of these variants for tolerance to aneuploidy. Among them, *SCH9* was particularly remarkable, since it was mutated at distinct loci in four different disomic lines (D10, D13, D2, D15). This kinase is involved in growth control through regulation of ribosome biogenesis and translation and has been previously implicated in the stabilization of tetraploidy^46^. We experimentally investigated two coding mutations in *SCH9* (Fig. 6). We observed that *SCH9* W377C improved proliferation in many disomic strains and thus likely represented a robust adaptation to aneuploidy. In contrast, *SCH9* F760L did not have a consistent effect on proliferation, suggesting that its fitness benefit, if present, is limited to a specific genetic background. Although the mechanism of *SCH9*-mediated proliferation improvement remains unclear, some insights come from earlier work. Previously, Pavelka et al.^21^ has shown that many aneuploid strains grew significantly better than euploid strains when treated with Rapamycin, which inhibits *TOR1* activity and prevents *TOR1*-mediated activation of *SCH9*. Based on this observation, we speculate that the adaptive mutations in *SCH9* may be inhibitory, reducing *SCH9*-mediated regulation of ribosome biogenesis and decreasing translation initiation. This could contribute to alleviate the proteotoxic stress associated with chromosome copy number alteration^49^. Furthermore, *SCH9* and its mammalian ortholog *S6K1* are known to integrate nutrient signals from *TOR1* with growth and stress signals from sphingolipids^50^. Also, it was shown that *ΔSCH9* yeast cells display decreased levels of ceramides^51^, and that the abundance of ceramide anti-correlates with proliferation of aneuploid cells^52^. Therefore, we consider regulation of sphingolipid levels as a potentially relevant mechanism of the *SCH9*-mediated response to aneuploidy, which prompts further investigation.

In addition, we identified one variant, *APN1* K352N, being recurrently fixed in all lines of disome D12. We believe that this mutation constitutes an adaptive event conferring enhanced fitness in aneuploid cells. We showed that *APN1* K352N enhances proliferation in the presence of oxidative DNA damage or alkylating agent MMS in a variety of genetic backgrounds (Fig. 5, Supplementary Fig. 9). However, perhaps surprisingly, the evolved lines of D12 did not have a lower rate of mutations than other disomes. It is possible that the fitness benefit of *APN1* K352N resides in a faster repair response, rather than higher efficiency. Alternatively, it is possible that *APN1* K352N indeed protects against mutations, but its effect is masked by a high mutator background in the disome D12. Altogether, our results show that our set-up can be used to identify beneficial mutations under specific forms of stress, such as aneuploidy. Their identification allows to pinpoint the most relevant biological processes for the evolutionary response to such stress. Besides, these mutations can now be used in directed evolution to improve fitness in stress conditions.

We performed a comprehensive analysis of the gene expression landscape of evolved disomic strains, comparing them with their disomic and WT ancestors. Our results indicate that, generally, evolved lines feature transcription profiles similar to their disomic ancestors, characterized by elevated expression levels of genes located on duplicated chromosomes (Fig. 7, Supplementary Fig. 13b). However, adaptive evolution had the effect of attenuating the largest transcript level alterations introduced by aneuploidy (Fig. 8a, Supplementary Fig. 13a). We thus focused on recurrent gene expression changes emerged throughout the evolution of multiple disomic strains. Functional enrichment of these genes pointed to pathways in common with those of recurrently mutated genes, so that our analyses converged in identifying gene networks central to the response to aneuploidy. During adaptive evolution, disomic strains tended to upregulate ribosome biogenesis and other translation-related factors, while downregulating amino acid biosynthesis (related to growth control and environmental stress response), endonucleases and endopeptidases (related to RNA and protein degradation), and stress response. Remarkably, we identified five TFs whose motifs are enriched in our gene set: *XBP1*, *MOT3*, *HSF1*, *LEU3*, and *GCR1*. This suggests that these factors may be implicated in the gene expression response observed in aneuploid lines during adaptive evolution. Additional lines of evidence also implicated some of these TFs: the expression level of *XBP1* and *GCR1* correlated across disomic lines with the degree of gene expression attenuation towards WT state, and deletion of *LEU3* improved proliferation in many disomic backgrounds. We attempted to establish direct causal relationships by introducing extra gene copies to increase TF expression in disomes, but this did not result in consistent fitness benefits, suggesting that complex post-translational TF regulation may also be relevant. While further investigation is required to validate and untangle their molecular mechanisms, our results implicate these TFs as potential modulators of adaptation to aneuploidy. It is thus useful to review their known functions and possible links with response to unbalanced karyotype burden.

*XBP1* is known to regulate transcription of endoplasmic reticulum stress-response genes, and its expression increases as a part of the unfolded protein response (UPR)^53^. A previous study reported that, even though disomic strains suffer from proteotoxic stress, they did not activate the UPR^49^. In contrast, our results suggest that the UPR is indeed activated in disomic lines during adaptive growth. *HSF1* is also linked to the UPR: it was shown to regulate *HSP90*-mediated protein folding, and increased levels of *HFS1* counteract the adverse effects of aneuploidy^54^. Furthermore, the activity of *HSF1* is known to be compromised in aneuploidy cells. Our discovery of *XBP1* and *HSF1* motifs in commonly regulated genes points to a key role of the UPR during adaptation to aneuploidy, presumably to handle proteotoxic stress. On the other hand, *GCR1* and *MOT3* are known to regulate glycolytic gene expression and glucose transporters. Several adaptive mutations in *MOT3* were shown to increase fitness of aneuploid cells^55^. The presence of binding motifs for *GCR1*, *MOT3*, as well as *LEU3* (which activates branched chain amino acid synthesis) points to potential strategies for metabolic adaptation.

Our study provides insights into how cells adapt to tolerate chromosomal imbalance, allowing to employ the characteristic genomic instability of aneuploidy for rapid adaptive stress responses, while attenuating its detrimental effects due to impaired gene dosage. Dissecting these mechanisms is essential for understanding the rapid adaptation of unicellular organisms to stress environments, which is often characterized by aneuploid states^56–58^. Notably, the same also applies to the evolution of cancers in humans, which are under constant stress from the immune system, scarce oxygen and ATP availability. Aneuploidy is believed to play a key role in enhancing stress-mediated adaptation and survival ability of cancer cells^59^. The disomic yeast strains we used in this work are haploid and sensitive to chromosome copy number alterations, so that it is unclear whether the mechanisms of aneuploidy tolerance highlighted here are relevant for other aneuploid genomes including cancer cells, which mostly start as diploid. In this regard, future research in near-haploid cancer cell lines^60,61^ may be informative.

## Methods

### Yeast strains and growth conditions

The W303 derivatives of yeast disomic strains were reported previously^6,18^. All experiments were performed in Yeast Nitrogen Based synthetic media supplemented with all amino acid except histidine (YNB-His) and supplied with 200 µg/ml Kanamycin (G418). This setup forces the maintenance of disomy, since each copy of the disomic chromosome harbors a selection cassette (*HIS3* and *KANMX*). The parental WT W303 strain was also grown under these conditions, as selection marker cassettes were integrated into its genome^6,18^. The laboratory evolution experiment was carried out by serial dilution. Single colonies of each disomic and WT cells were grown overnight at 30 °C with moderate shaking and then diluted by a factor of 1:500 with fresh YNB-His medium into three individual cultures to generate evolved lines for each strain. These lines were diluted daily by a factor of 1:500 (∼9 generations per dilution for WT cells) for 134 days. This procedure was repeated for a course of an estimated 1200 generations for WT, and aneuploid lines were grown for the same time duration. Due to their slower growth rate, this corresponded to fewer than 1200 generations, so that mutation rates are slightly underestimated for aneuploid lines. At the end of experimental evolution, whole populations were used to isolate DNA and RNA for genome and transcriptome sequencing.

### Growth assays and analysis of doubling time

Cells were grown at 30 °C, and OD_600_ measurements were taken at 15 min intervals using a Biotek-Epoch2 instrument until cells reached early stationary phase. Doubling time was calculated with R script by analyzing fitting spline function from growth curve slopes^62^. The maximum slope of the spline fit was used as an estimate for the growth rate and doubling time for each evolved line.

### Validating the function of *APN1* and *SCH9* mutations

The *APN1* gene was amplified with PCR from S288c genome and cloned into pBluescript SK (+) plasmid at the *SpeI-XhoI* restriction sites. This construct was used to introduce K352N mutation by PCR, and mutation was verified by Sanger sequencing. Both mutated and non-mutated *APN1* was cut from pBluescript SK (+) and ligated into low copy p413ADH vector containing the *HIS3* and hygromycin (*HYG*) selection markers, with *APN1* expression controlled by constitutive ADH promoter. Both *ΔAPN1* cells from yeast knockout library and Δ7 cells were transformed with plasmids to express mutated *APN1*, non-mutated *APN1* or an empty vector as control. Clones were selected on YNB -HIS, +HYG plates and grown overnight in liquid YNB *-HIS*, *+HYG* medium. On the next day, the OD was adjusted to 0.5, and 10-fold dilutions were spotted on YNB plates to test for resistance of *ΔAPN1* cells to various concentration of MMS. Viability of Δ7 cells was also tested with both spot assay and growth measurement. Plates were incubated for 4 days at 30 °C and pictured. A region encompassing 1 kb upstream and 250 bp downstream of the *SCH9* gene was amplified with PCR from S288c genome and cloned into p42Nat 1-F GW (https://www.addgene.org/58549/) plasmid, which harbors *natMX6* cassette for Natamycin resistance in yeast. This construct was used to introduce W377C and F760L mutations individually. Both mutated and non-mutated *SCH9* were amplified along with *natMX6* cassette, and in total 6 kb PCR fragments were used for replacement of the endogenous *SCH9* locus in WT and disomic strains. Colonies were selected on YNB -HIS, +NAT plates after 4 days and integration of PCR fragment was verified by PCR and Sanger sequencing. Individual colonies were grown overnight from each strain to test the effect of introduced mutation on proliferation next day.

### Genome and RNA-sequencing and data analysis

Genomic DNA libraries were prepared using NextEra genomic DNA kit according to the manufacturer’s manual. Sequencing of libraries were performed on the Illumina Hi-Seq platform with 75 bp paired-end read option. PFGE was conducted using a BioRad Contour-clamped homogeneous electric field (CHEF) Mapper XA system and genomic samples were prepared using the CHEF genomic DNA plug kit. Agarose-embedded chromosomal DNA preparation and running conditions were performed according to the kit manual.

Genomic reads were mapped using Bowtie 2 v2.1.0^63^ and the recently published high-quality genome for the W303 laboratory strain (GenBank: LYZE00000000.1) was used as a reference^64^. We used the Genome Analysis Toolkit (GATK)^65^ to calculate the depth of coverage for each chromosome. The “DepthOfCoverage” tool was applied to sorted *.bam files to generate a genome-wide table of sequencing depth for karyotype analysis. The average depth of coverage was 50x per strain; however, single evolved lines of disomes D10 and D13 were covered below 10x and were eliminated from further analyses. In addition, single lines of D14 and D16 were also eliminated due to sample contamination during growth. We normalized coverage by the total number of reads per sample. The genomic read densities were plotted with the ggplot2 package within the R statistical environment (https://www.r-project.org/). In addition, the ShortRead package^66^ was used to identify chromosome breakpoints, which were then verified by manually visualizing bam files on Tablet software. Mutations were analyzed with both GATK^65^ and Free Bayes^67^. Mutations identified by both software packages were considered for further analysis, and some of the mutations were verified by analyzing bam files with the Tablet software as well^68^. Mutations on duplicated chromosomes were analyzed with GATK -L option with at least 5 paired reads covering the corresponding mutation loci. Mutations were called if ≥40 and 80% reads mapped to the mutation on the duplicated and non-duplicated chromosomes, respectively, and mutations commonly identified in every ancestral strain and the corresponding evolved lines were eliminated, since these mutations accumulated in the ancestral strain prior to the evolution experiment.

RNA-seq libraries were prepared with using Cell-Seq2 method^69^. RNA-seq (FASTQ) files were mapped to the yeast reference genome; Ensembl build R64-1-1 was downloaded from the Illumina iGenome resource at support.illumina.com/sequencing/sequencing_software/igenome.html. Mapping was performed using Bowtie 2 v2.1.0^53^ and read counts per gene were calculated using Feature Counts^70^. To filter out genes with the low number of reads, only the genes with at least 1 count per million (cpm) in at least half of the strains were retained, resulting in an expression set of 6059 genes across 32 evolved lines (2 evolved lines from D14 and D16 were eliminated from the analysis due to sample contamination during library preparation and growth). Filtered data was then subjected to RLE normalization^71^. Differential gene expression analysis was performed using the R package EdgeR^72^, with FDR of 10% to increase the statistical power of subsequent functional enrichment analyses. When searching for genes with common expression changes across all evolved strains, a single statistical model with factorial variable representing shift from the ancestor to evolved strains was used. When searching for genes associated with a transcriptional shift towards WT, a single statistical model with numeric variable equal to the expression distance from the ancestor strain towards WT (see below) for each evolved strain was used. FDR threshold was set to 0.1^73^.

### Analysis of mutation rates and mutation distribution

We compared the variants predicted in each evolved disomic line and WT cells to the corresponding ancestral strains. The common mutations identified in both ancestral and evolved lines were eliminated to identify unique de novo mutations in the evolved strains. Apparent mutation rate was calculated by dividing the number of detected de novo mutations by the total number of bases in the reference genome sequence and by the number of generations that each strain was propagated. Mutation rates for the different mutation types were calculated by further normalizing mutations from AT base pairs by the AT fraction of the genome and mutations from GC base pairs by the GC fraction of the genome. Standard errors for apparent mutation rates were calculated as the square root of u/(n*g), where *u* is the estimated rate, *n* is the number of bases in the genome, and *g* is the number of generations^74^. The distribution and relationship with replication timing of mutations were analyzed according to a previously described method^75^.

### Annotation of mutations and gene enrichment analysis

Mutations on protein coding genes were annotated as either missense, nonsense or silent using SnpEff (http://snpeff.sourceforge.net/). Intergenic (non-coding) mutations were attributed to the promoters of their closest gene if their transcription start sites were located within 750 bp with a custom Python script (this threshold was chosen based on a previous analysis of the average yeast promoter length^32^. Gene enrichment (GO) analysis was performed using gprofiler tool^76^. TF binding motif search and related TF network were generated using yeastract tools^77^.

### Gene expression distance metric

We formulated a custom distance metric to evaluate the gene expression dynamics of disomic cells throughout adaptive evolution. We calculated this between WT and each disomic strain (ancestors and evolved lines separately) as the Euclidean distance of their expression levels in log space (i.e., square root of sum of square differences of the log of values). To focus on changes relevant for aneuploidy, at each comparison we considered only those genes that were altered in the ancestral disomes, using a threshold of absolute fold change ≥ 1.5 (for evolved disomic lines, we considered the same gene set as their corresponding ancestor strains). We then normalized the distance between WT and each evolved disomic line by the distance between WT and their corresponding ancestor disome. This resulted in the “normalized distance to WT” values shown in Supplementary Fig. 10a. For practical purposes, we then reformulated this as “normalized shift towards WT” (shown in Fig.6), equal to 1 min the distance as just defined. Positive shift values indicate that the largest gene expression differences between ancestor disomic lines and WT were attenuated during adaptive evolution.

### Statistical analysis

All statistical analyses were performed in the R statistical environment (https://www.r-project.org/). We used a two-sided binomial test to assess the significance of the differential distribution of mutations (substitutions and indels) in coding and non-coding regions. The number of mutations in target regions was considered as the number of successes, whereas the total number of mutations was considered as the number of trials. The probability of success was set to the proportion of the target sequences in the genome (74% for coding regions). We used the same approach to test if the fraction of mutations were preferentially located on the duplicated chromosome. Probabilities under null hypothesis were calculated based on the ratio of the corresponding chromosome length to the sum of lengths of all chromosomes. Obtained p-values were adjusted for multiple comparisons by the Benjamini-Hochberg procedure.

### Reporting summary

Further information on research design is available in the Nature Research Reporting Summary linked to this article.

## Supplementary information

Supplementary Information

Reporting Summary

Description of Additional Supplementary Files

Supplementary Data 1

Supplementary Data 2

Supplementary Data 3

Supplementary Data 4

Supplementary Data 5

Supplementary Data 6

## Data Availability

The genome reads reported in this study are deposited in the BioProject database, www.ncbi.nlm.nih.gov/bioproject (accession no. PRJNA478560). Transcriptome (RNA-sequencing) reads and fragments per kilobase of transcript per million mapped read values are deposited in the Gene Expression Omnibus (GEO) database, www.ncbi.nlm.nih.gov/geo (accession no. GSE119272). All other data analyzed during this study are included in this article and its Supplementary Information files. The source data underlying Figs. 1a, 3c, 4a, b and Supplementary Fig. 15a, b are provided as a Source Data file. The source data for Fig. 6 and Supplementary Fig. 15C are provided in Supplementary Data 3 and Supplementary Data 6.

## Source data

Source Data

## Code availability

The custom Python script called “get_overlapping_features.py” and its description for attributing intergenic (non-coding) mutations to promoters of their closest genes were uploaded to https://github.com/marco-mariotti/genomic_tools.

## References

[1] Siegel JJ, Amon A (2012). New insights into the troubles of aneuploidy. Annu. Rev. Cell. Dev. Biol..

[2] Santaguida S, Amon A (2015). Short- and long-term effects of chromosome mis-segregation and aneuploidy. Nat. Rev. Mol. Cell Biol..

[3] Maurer M (2015). Chromosomal aneuploidies and early embryonic developmental arrest. Int. J. Fertil. Steril..

[4] Sheltzer JM (2011). Aneuploidy drives genomic instability in yeast. Science.

[5] Sunshine AB (2016). Aneuploidy shortens replicative lifespan in *Saccharomyces cerevisiae*. Aging Cell..

[6] Torres EM (2007). Effects of aneuploidy on cellular physiology and cell division in haploid yeast. Science.

[7] Torres EM (2010). Identification of aneuploidy-tolerating mutations. Cell.

[8] Taylor AM (2018). Genomic and functional approaches to understanding cancer aneuploidy. Cancer Cell.

[9] Chen G, Rubinstein B, Li R (2012). Whole chromosome aneuploidy: big mutations drive adaptation by phenotypic leap. Bioessays.

[10] Chen G (2015). Targeting the adaptability of heterogeneous aneuploids. Cell.

[11] Selmecki AM (2015). Polyploidy can drive rapid adaptation in yeast. Nature.

[12] Millet C, Makovets S (2016). Aneuploidy as a mechanism of adaptation to telomerase insufficiency. Curr. Genet..

[13] Yona AH (2012). Chromosomal duplication is a transient evolutionary solution to stress. Proc. Natl Acad. Sci. USA.

[14] Kaya A (2015). Adaptive aneuploidy protects against thiol peroxidase deficiency by increasing respiration via key mitochondrial proteins. Proc. Natl Acad. Sci. USA.

[15] Linder RA, Greco JP, Seid lF, Matsui T, Ehrenreich IM (2017). The stress-inducible peroxidase TSA2 underlies a conditionally beneficial chromosomal duplication in *Saccharomyces cerevisiae*. G3 (Bethesda).

[16] Selmecki AM, Dulmage K, Cowen LE, Anderson JB, Berman J (2009). Acquisition of aneuploidy provides increased fitness during the evolution of antifungal drug resistance. PLoS Genet..

[17] Huettel B, Kreil DP, Matzke M, Matzke AJ (2008). Effects of aneuploidy on genome structure, expression, and interphase organization in *Arabidopsis thaliana*. PLoS Genet..

[18] Sheltzer JM, Torres EM, Dunham MJ, Amon A (2012). Transcriptional consequences of aneuploidy. Proc. Natl Acad. Sci. USA..

[19] Stingele S (2012). Global analysis of genome, transcriptome and proteome reveals the response to aneuploidy in human cells. Mol. Syst. Biol..

[20] Dephoure N (2014). Quantitative proteomic analysis reveals posttranslational responses to aneuploidy in yeast. eLife.

[21] Pavelka N (2010). Aneuploidy confers quantitative proteome changes and phenotypic variation in budding yeast. Nature.

[22] Zhu YO, Sherlock G, Petrov DA (2016). Whole genome analysis of 132 clinical *Saccharomyces cerevisiae* strains reveals extensive ploidy variation. G3.

[23] Peter J (2018). Genome evolution across 1,011 Saccharomyces cerevisiae isolates. Nature.

[24] Hose J (2015). Dosage compensation can buffer copy-number variation in wild yeast. eLife.

[25] Tan Z (2013). Aneuploidy underlies a multicellular phenotypic switch. Proc. Natl Acad. Sci. USA.

[26] Kokina A, Kibilds J, Liepins J (2014). Adenine auxotrophy–be aware: some effects of adenine auxotrophy in Saccharomyces cerevisiae strain W303-1A. Fems. Yeast. Res..

[27] Nevzglyadova OV (2011). The effect of red pigment on the amyloidization of yeast proteins. Yeast.

[28] Bouchonville K, Forche A, Tang KE, Selmecki A, Berman J (2009). Aneuploid chromosomes are highly unstable during DNA transformation of *Candida albicans*. Eukaryot. Cell.

[29] Zhu J, Pavelka N, Bradford WD, Rancati G, Li R (2012). Karyotypic determinants of chromosome instability in aneuploid budding yeast. PLoS Genet..

[30] Lynch M (2008). A genome-wide view of the spectrum of spontaneous mutations in yeast. Proc. Natl Acad. Sci. USA.

[31] Passerini V (2016). The presence of extra chromosomes leads to genomic instability. Nat. Commun..

[32] Cherry JM (1997). Genetic and physical maps of *Saccharomyces cerevisiae*. Nature.

[33] Serero A, Jubin C, Loeillet S, Legoix-Ne P, Nicolas AG (2014). Mutational landscape of yeast mutator strains. Proc. Natl Acad. Sci. USA.

[34] Zhu YO, Siegal ML, Hall DW, Petrov DA (2014). Precise estimates of mutation rate and spectrum in yeast. Proc. Natl Acad. Sci. USA.

[35] Kino K, Sugiyama H (2001). Possible cause of G-C–>C-G transversion mutation by guanine oxidation product, imidazolone. Chem. Biol..

[36] Alexandrov LB (2013). Signatures of mutational processes in human cancer. Nature.

[37] Pan Hl, Ma P, Zhu W, Schultz RM (2008). Age-associated increase in aneuploidy and changes in gene expression in mouse eggs. Dev. Biol..

[38] Fragouli E (2015). Altered levels of mitochondrial DNA are associated with female age, aneuploidy, and provide an independent measure of embryonic implantation potential. PLoS Genet..

[39] Tao X, Landis JN, Scott RT, Treff N, Lonczak A (2016). Mitochondrial DNA content is increased in the aneuploid mouse blastocyst. Fertil. Steril..

[40] Beier H, Grimm M (2001). Misreading of termination codons in eukaryotes by natural nonsense suppressor tRNAs. Nucleic Acids Res..

[41] Pearson D (1985). Mutations preventing expression of sup3 tRNASer nonsense suppressors of *Schizosaccharomyces pombe*. Mol. Cell. Biol..

[42] Ramotar D, Popoff SC, Gralla EB, Demple B (1991). Cellular role of yeast Apn1 apurinic endonuclease/3’-diesterase: repair of oxidative and alkylation DNA damage and control of spontaneous mutation. Mol. Cell. Biol..

[43] Ma W, Resnick MA, Gordenin DA (2008). Apn1 and Apn2 endonucleases prevent accumulation of repair-associated DNA breaks in budding yeast as revealed by direct chromosomal analysis. Nucleic Acids Res..

[44] Ishchenko AA, Yang X, Ramotar D, Saparbaev M (2005). The 3′→5′ Exonuclease of Apn1 provides an alternative pathway to repair 7,8-Dihydro-8-Oxodeoxyguanosine in *Saccharomyces cerevisiae*. Mol. Cell. Biol..

[45] Kaya A (2014). Thiol peroxidase deficiency leads to increased mutational load and decreased fitness in *Saccharomyces cerevisiae*. Genetics.

[46] Lu YJ, Swamy KB, Leu JY (2016). Experimental evolution reveals interplay between Sch9 and polyploid stability in yeast. PLoS Genet..

[47] Dodgson SE (2016). Chromosome-specific and global effects of aneuploidy in *Saccharomyces cerevisiae*. Genetics.

[48] Ronen M, Botstein D (2006). Transcriptional response of steady-state yeast cultures to transient perturbations in carbon source. Proc. Natl Acad. Sci. USA.

[49] Oromendia AB, Dodgson SE, Amon A (2012). Aneuploidy causes proteotoxic stress in yeast. Genes Dev..

[50] Huang X, Liu J, Dickson RC (2012). Down-regulating sphingolipid synthesis increases yeast lifespan. PLoS Genet..

[51] Swinnen E (2014). The protein kinase Sch9 is a key regulator of sphingolipid metabolism in *Saccharomyces cerevisiae*. Mol. Biol. Cell.

[52] Tang YC, Williams BR, Siegel JJ, Amon A (2011). Identification of aneuploidy-selective antiproliferation compounds. Cell.

[53] Clarke R (2012). Endoplasmic reticulum stress, the unfolded protein response, autophagy, and the integrated regulation of breast cancer cell fate. Cancer Res..

[54] Donnelly N, Passerini V, Dürrbaum M, Stingele S, Storchová Z (2014). HSF1 deficiency and impaired HSP90-dependent protein folding are hallmarks of aneuploid human cells. EMBO J..

[55] Scott AL, Richmond PA, Dowell RD, Selmecki AM (2017). The Influence of polyploidy on the evolution of yeast grown in a sub-optimal carbon source. Mol. Biol. Evol..

[56] Gerstein AC, Berman J (2015). Shift and adapt: the costs and benefits of karyotype variations. Curr. Opin. Microbiol..

[57] Hope EA, Dunham MJ (2014). Ploidy-regulated variation in biofilm-related phenotypes in natural isolates of *Saccharomyces cerevisiae*. G3 (Bethesda).

[58] Hirakawa MP (2015). Genetic and phenotypic intra-species variation in *Candida albicans*. Genome Res..

[59] Holland AJ, Cleveland DW (2012). Losing balance: the origin and impact of aneuploidy in cancer. EMBO Rep..

[60] Oshimura M, Freeman AI, Sandberg AA (1977). Chromosomes and causation of human cancer and leukemia. XXIII. Near-haploidy in acute leukemia. Cancer.

[61] Lawrence B (2018). Recurrent loss of heterozygosity correlates with clinical outcome in pancreatic neuroendocrine cancer. NPJ Genom. Med..

[62] Kahm M, Hasenbrink G, Lichtenberg-Fraté H, Ludwig J, Kschischo M (2010). grofit: Fitting biological growth curves with R. J. Stat. Softw.

[63] Langmead B, Salzberg SL (2012). Fast gapped-read alignment with Bowtie 2. Nat. Methods.

[64] Matheson K, Parsons L, Gammie A (2017). Whole-Genome sequence and variant analysis of W303, a widely-used strain of *Saccharomyces cerevisiae*. G3 (Bethesda).

[65] McKenna A (2010). The Genome Analysis Toolkit: a MapReduce framework for analyzing next-generation DNA sequencing data. Genome Res..

[66] Morgan M (2009). ShortRead: a bioconductor package for input, quality assessment and exploration of high-throughput sequence data. Bioinformatics.

[67] Garrison, E. & Marth, G. Haplotype-based variant detection from short-read sequencing. Preprint at https://arXiv.org/abs/1207.3907 (2012).

[68] Milne I (2013). Using tablet for visual exploration of second-generation sequencing data. Brief. Bioinform..

[69] Hashimshony T (2016). CEL-Seq2: sensitive highly-multiplexed single-cell RNA-Seq. Genome Biol..

[70] Liao Y, Smyth GK, Shi W (2014). featureCounts: an efficient general-purpose program for assigning sequence reads to genomic features. Bioinformatics.

[71] Maza E, Frasse P, Senin P, Bouzayen M, Zouine M (2013). Comparison of normalization methods for differential gene expression analysis in RNA-Seq experiments: a matter of relative size of studied transcriptomes. Commun. Integr. Biol..

[72] Robinson MD, McCarthy DJ, Smyth GK (2010). edgeR: a bioconductor package for differential expression analysis of digital gene expression data. Bioinformatics.

[73] Benjamini Y, Hochberg Y (1995). Controlling the false discovery rate: a practical and powerful approach to multiple testing. J. R. Stat. Soc. B.

[74] Denver DR (2004). High mutation rate and predominance of insertions in the *Caenorhabditis elegans* nuclear genome. Nature.

[75] Koren A, Soifer I, Barkai N (2010). MRC1-dependent scaling of the budding yeast DNA replication timing program. Genome Res..

[76] Reimand J (2016). g:Profiler-a web server for functional interpretation of gene lists (2016 update). Nucleic Acids Res..

[77] Teixeira MC (2018). YEASTRACT, an upgraded database for the analysis of transcription regulatory networks in *Saccharomyces cerevisiae*. Nucleic Acids Res..

