# Supplementary material for: Molecular signatures of aneuploidy-driven adaptive evolution
Source: Nat Commun. 2020 Jan 30;11:588. doi: 10.1038/s41467-019-13669-2 (PMC6992709; doi:10.1038/s41467-019-13669-2)
Supplement: Supplementary file 1 — Supplementary Information [file 41467_2019_13669_MOESM1_ESM.pdf]

SUPPLEMENTARY INFORMATION

Molecular Signatures of Aneuploidy-Driven Adaptive Evolution

Kaya et al.

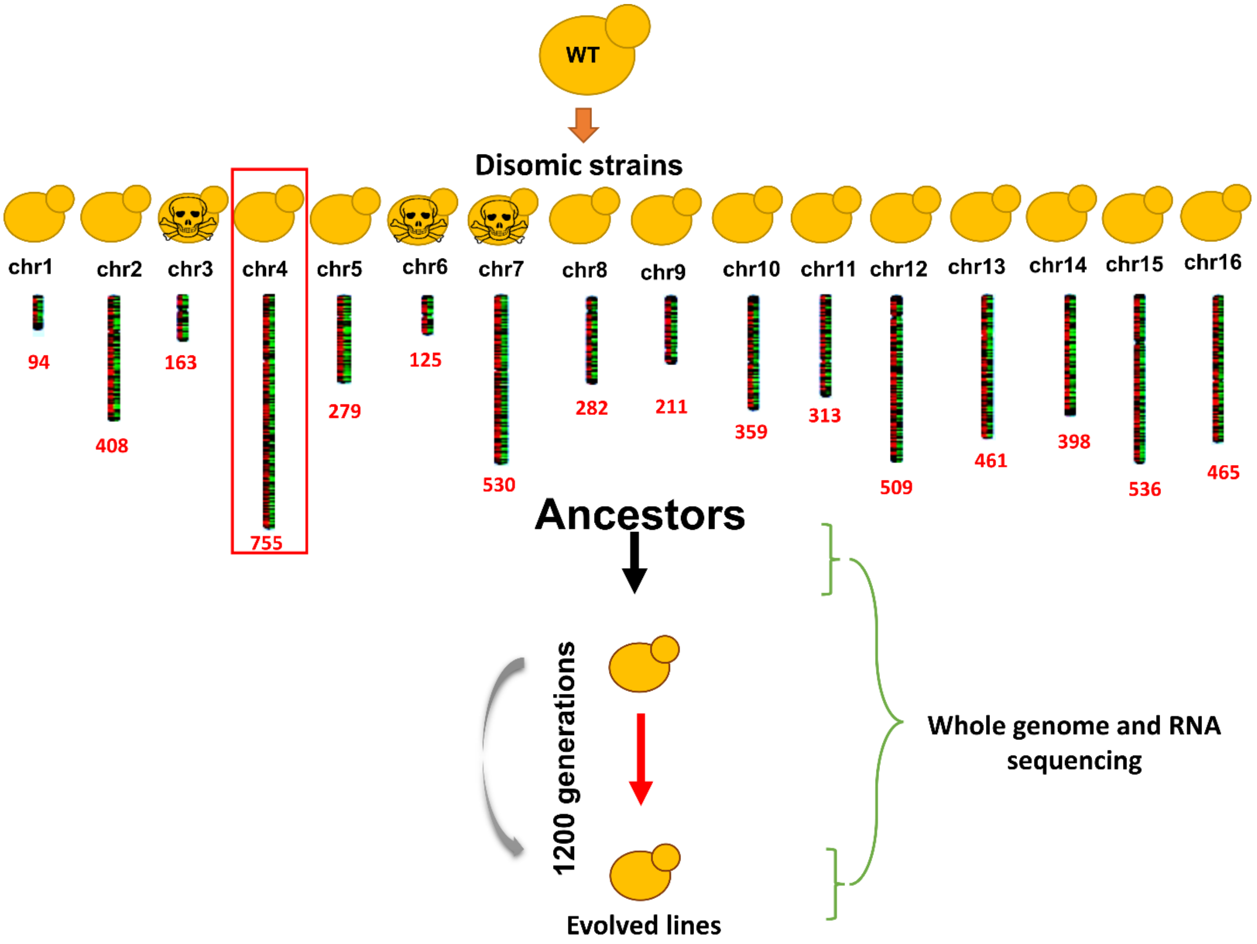

Supplementary Figure 1. Experimental design of the laboratory evolution experiment. Disomic strains were generated in W303 WT background. Duplication of chromosomes III, VI, and VII was lethal, and chromosome IV disomic strains grew very slowly, so they were excluded from the experiment. For the remaining strains, we started each with several mutation accumulation lines and subjected them to a bottleneck process over the course of 1,200 generations, followed by genome sequencing and RNA sequencing of the original and evolved lines. The number of genes localized on each chromosome is indicated with red numbers.

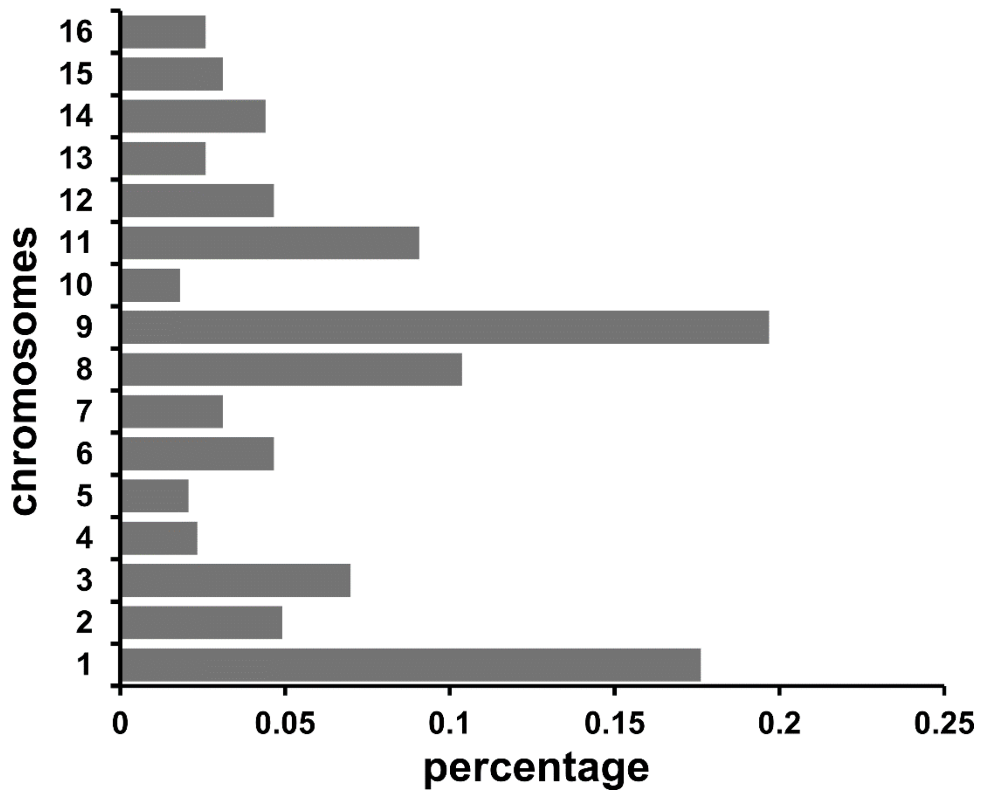

Supplementary Figure 2. Frequency of chromosome duplication for 16 yeast chromosomes across 386 wild yeast isolates. Bar plots represent percentage of observed duplication for each chromosome. Aneuploidy status of these WT strains was published previously (1, 2).

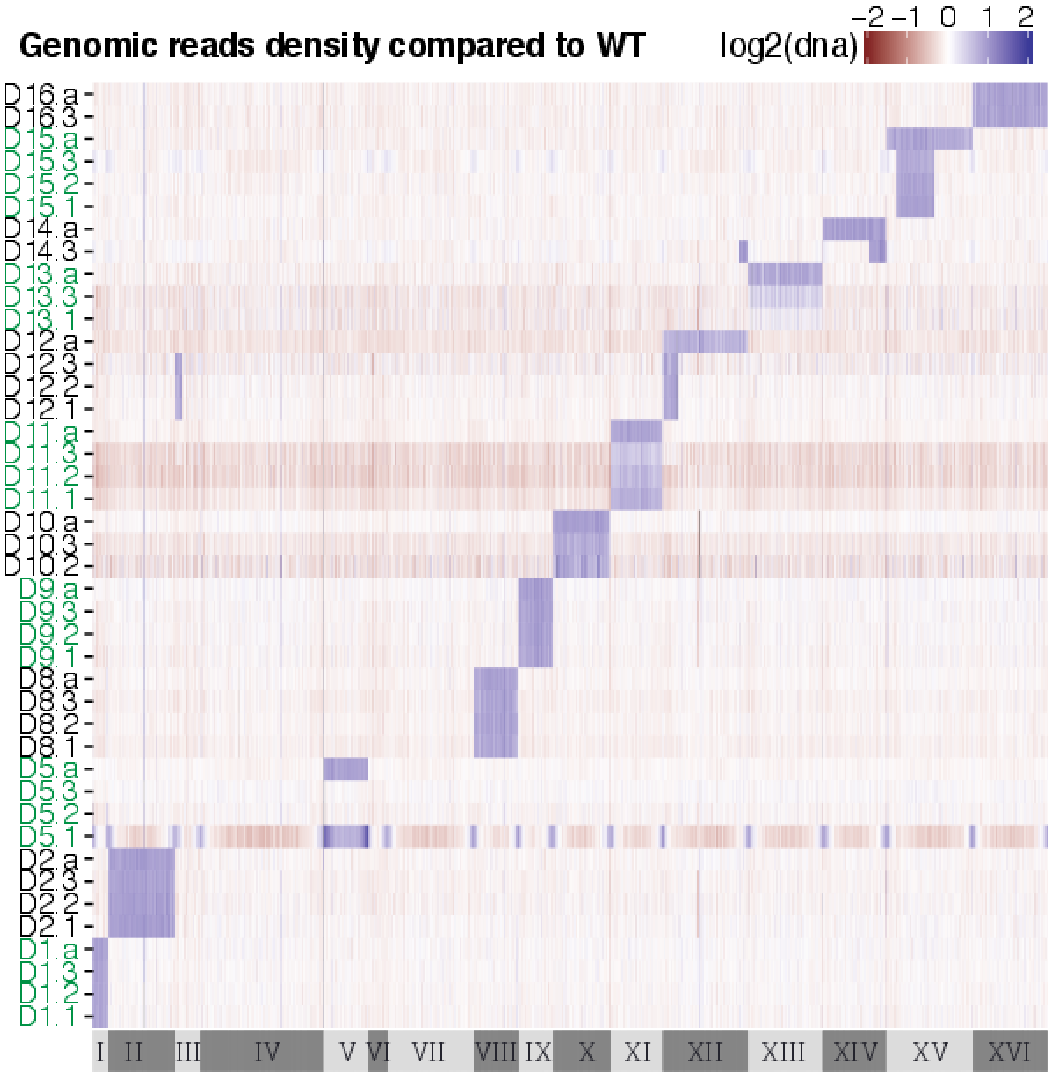

Supplementary Figure 3. Karyotype profiles of ancestor and evolved disomic lines based on genomic read density. Heatmap representing genomic reads density per gene normalized as in Fig. 2. Blue and red regions have increased and decreased read density compared to WT, respectively. The bottom map shows the chromosomal localization of each gene.

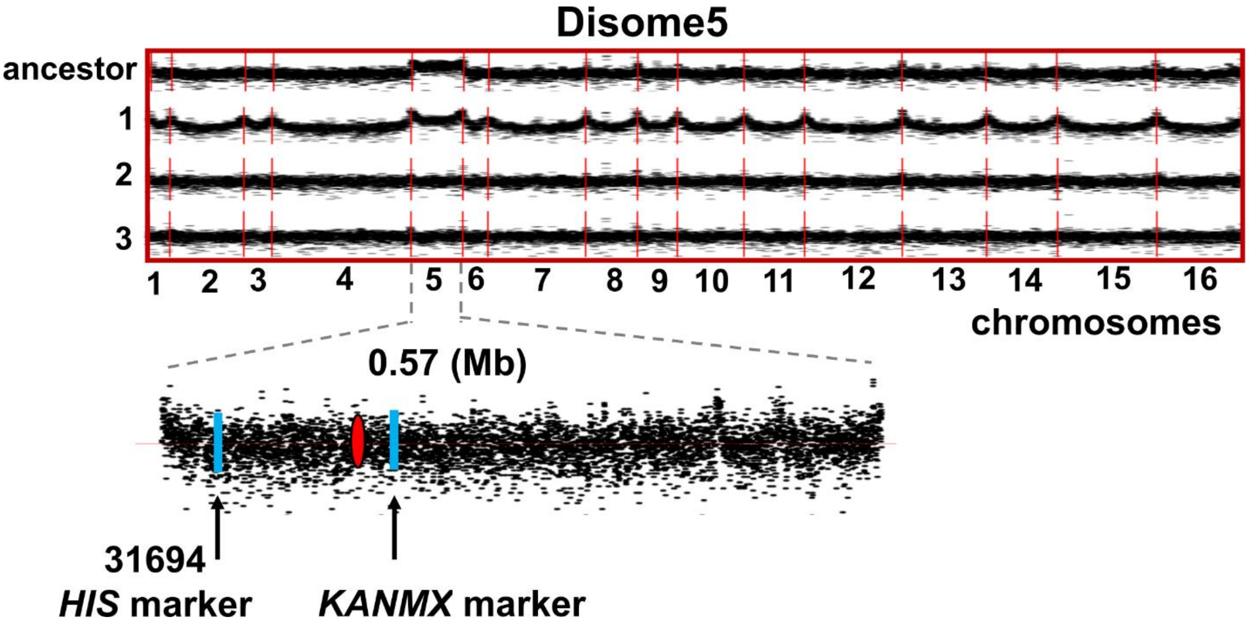

Supplementary Figure 4. Karyotype of disome D5. Genomic read density for evolved and ancestral lines of this disome is shown throughout the genome. Karyotype was analyzed as described in the main text. Lower panel shows chromosome V in one of the evolved lines with a monosomic state. *HIS* and *KANMX* markers are shown in blue, and the centromere in red.

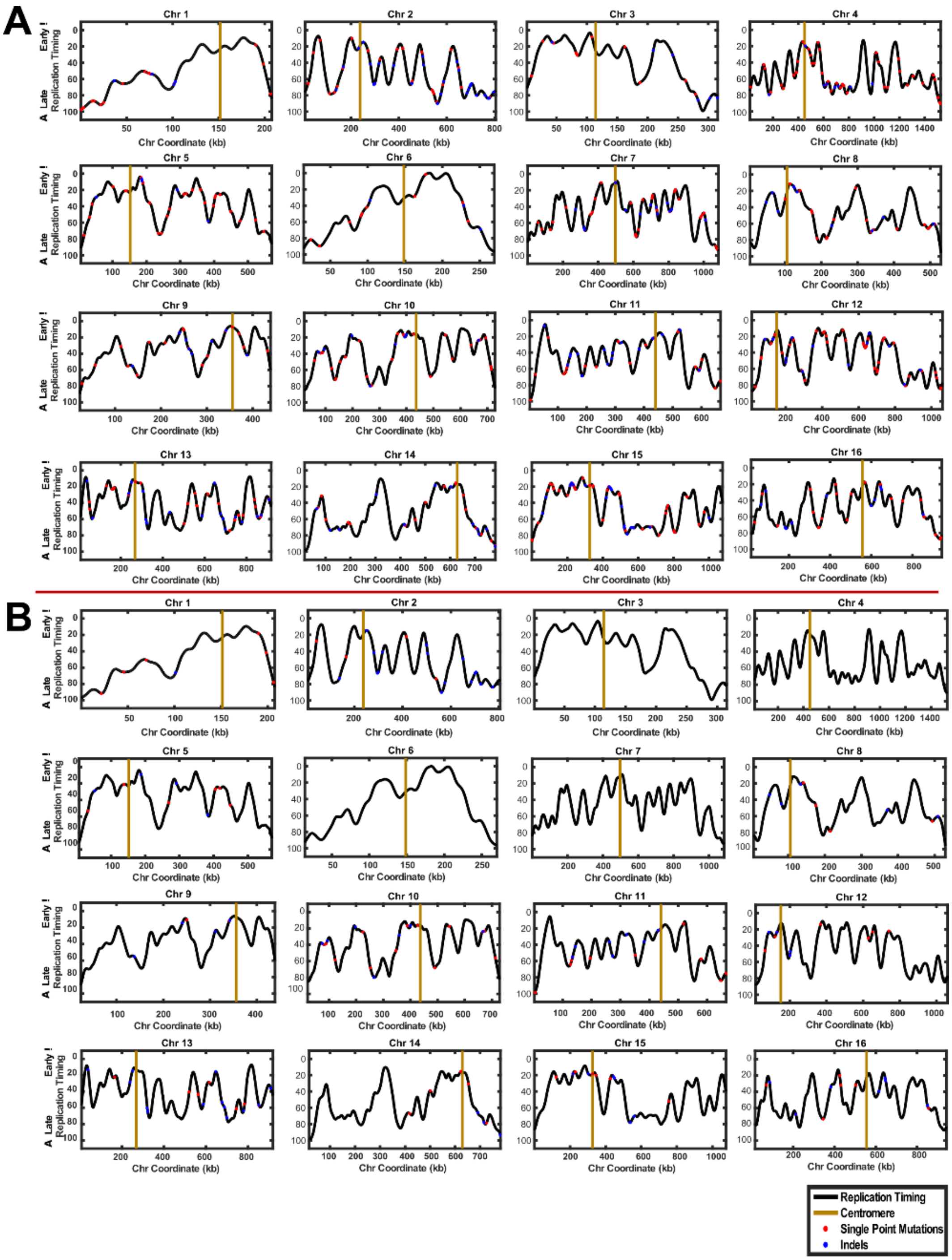

Supplementary Figure 5. Relationship between mutations in evolved disomic lines and replication timing. (A) Replication timing of all mutations observed during adaptive growth of disomic strains. (B) Replication timing of mutations mapped to the duplicated chromosomes observed during adaptive growth of disomic strains.

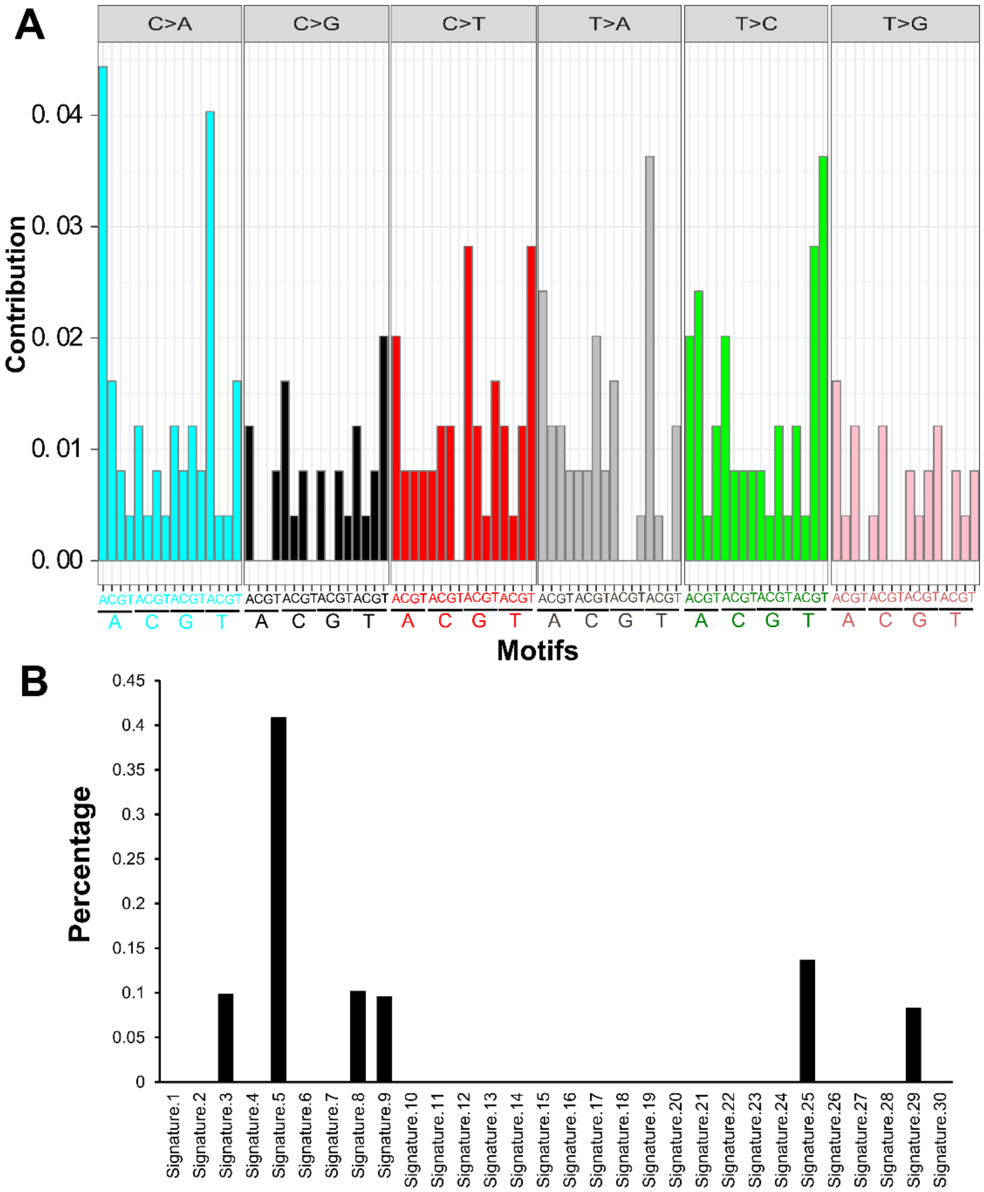

Supplementary Figure 6. Cosmic signatures of genome-wide mutation patterns in disomic strains. (A) Contribution of each nucleotide changes in all disomic strains is displayed using the six substitution subtypes: C>A, C>G, C>T, T>A, T>C, and T>G using R Bioconductor package "BSgenome.Scerevisiae.UCSC.sacCer3". **(B)** R package "deconstructSigs" was used to analyze the putative contribution of cancer mutational signatures (as described in https://cancer.sanger.ac.uk/cosmic/signatures/SBS/) to the observed mutations in disomic yeast.

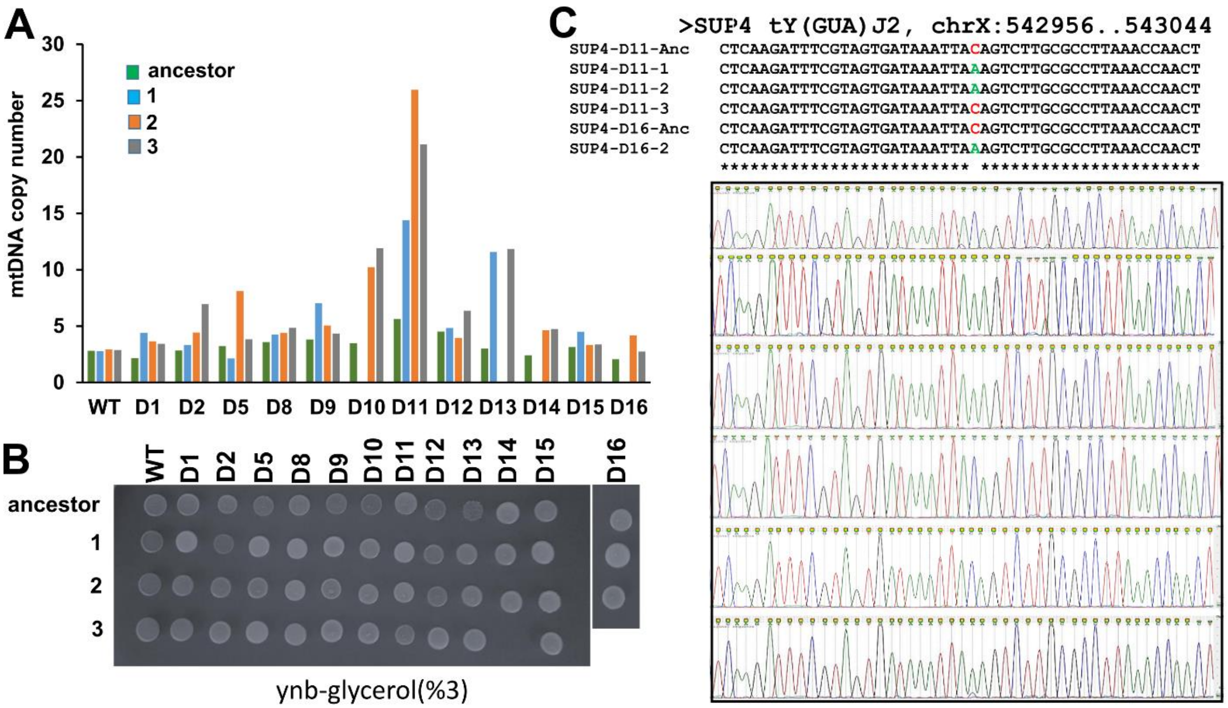

Supplementary Figure 7. Mitochondrial DNA (mtDNA) copy number and respiratory growth of evolved lines. (A) Each bar represents the mtDNA copy number of ancestor and evolved lines. mtDNA copy number was computed as the average mitochondrial genome coverage divided by the average nuclear genome coverage. (B) Respiratory growth of ancestor and evolved lines tested on YNB medium containing 3% glycerol as a carbon source. All strains grew colonies in this media, indicating that none was respiratory deficient. (C) Examples of Sanger sequencing results of *SUP4* Tyr-tRNA. Sequences were amplified by PCR from the genomic DNA of corresponding strains, and cleaned PCR fragments were sequenced.

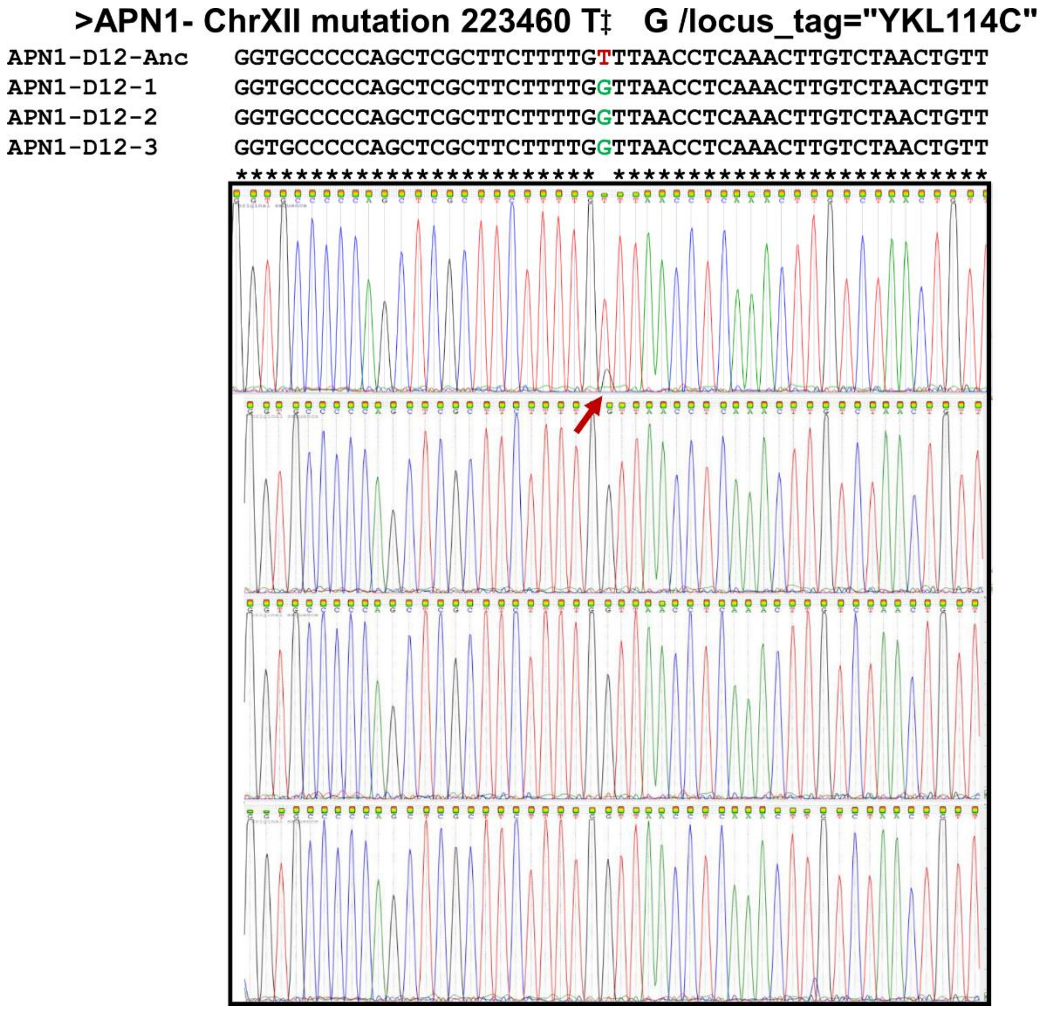

Supplementary Figure 8. Sanger sequencing of APN1. *APN1* was amplified by PCR from the genomic DNA of the corresponding strains, and cleaned PCR fragments were sequenced. Red arrow points to the location, whose analysis revealed that the T→G variant was already present at low frequency in the ancestor disome D12 strain.

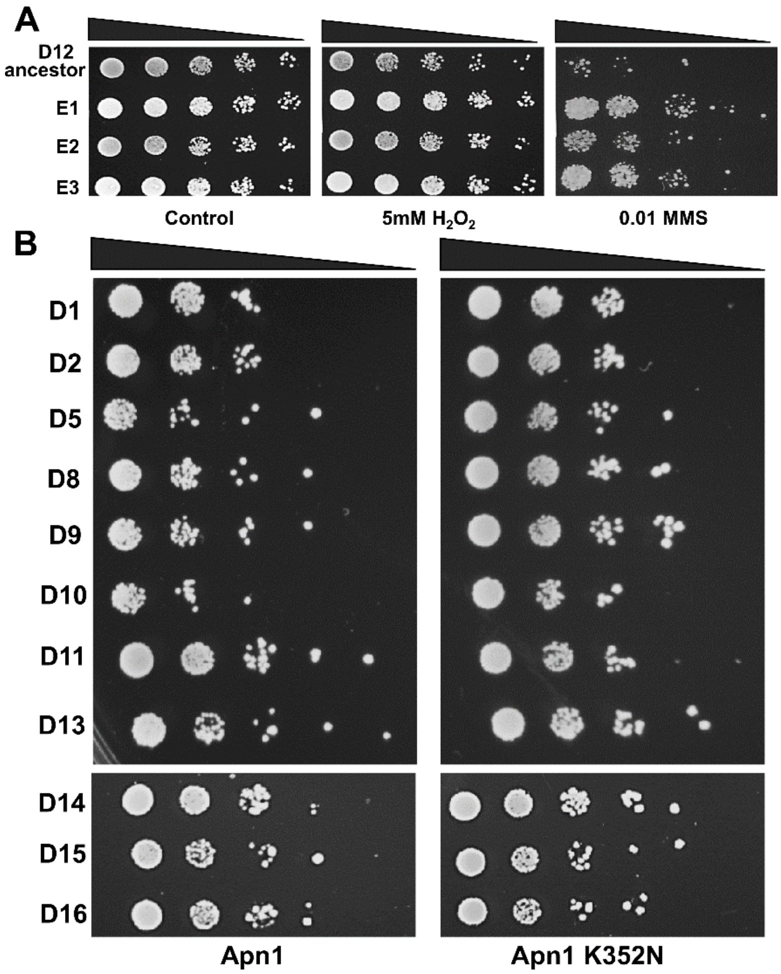

Supplementary Figure 9. Effect of Apn1 K352N mutation on survival of disomic strains under genotoxic stress. (A) Spot assay to assess the survival effect of Apn1 Lys352Asn mutation under conditions of H_2O_2 and 0.01 MMS (%) stress in ancestor and evolved D12 disomic strains. Cells (OD600 = 0.6) were pretreated with 5 mM H_2O_2 for 30 min and spotted with 10-fold dilution onto YNB -HIS plates. The plates were supplemented with 0.01% MMS at 10-fold dilution to test viability under MMS stress. **(B)** Spot assay for survival effect of *Apn1* K352N mutation under conditions of MMS stress across different disomic strains. Plates with YNB medium without His were supplemented with 0.01% MMS, and disomic strains harboring the vector expressing endogenous Apn1 or Apn1 K352N were spotted with 10-fold dilution onto plates.

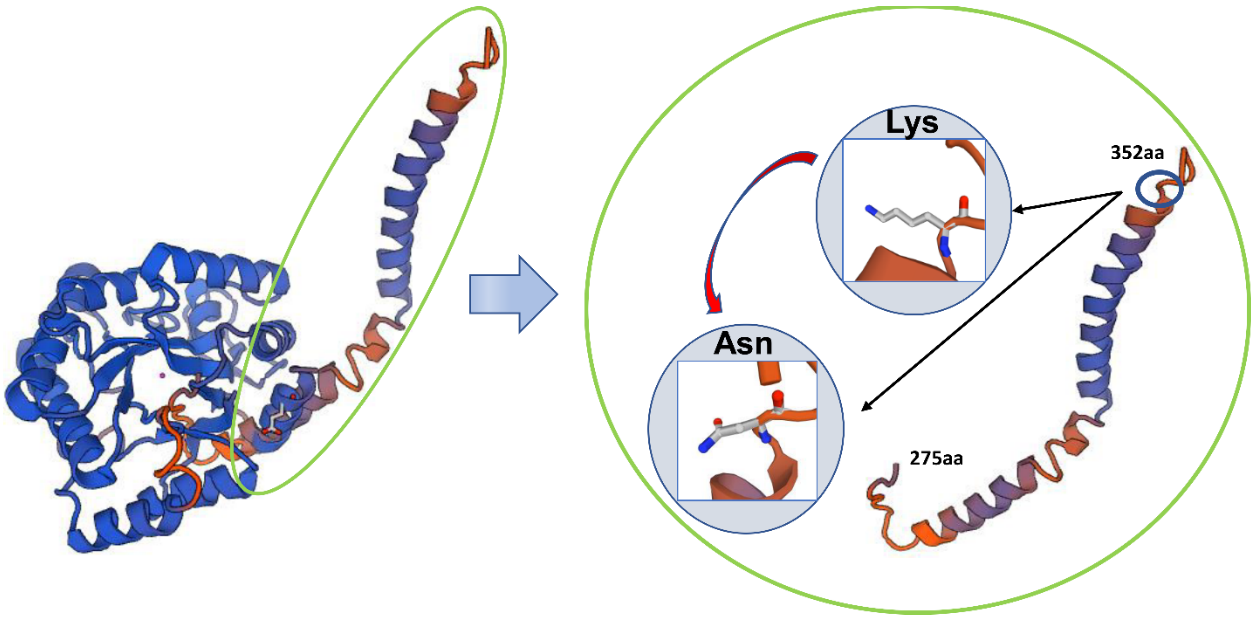

Supplementary Figure 10. Modeling of the APN1 structure with mutated residue 352. Structure of Apn1 with the mutated residue 352 was obtained by using I-TASSER; (https://zhanglab.ccmb.med.umich.edu/).

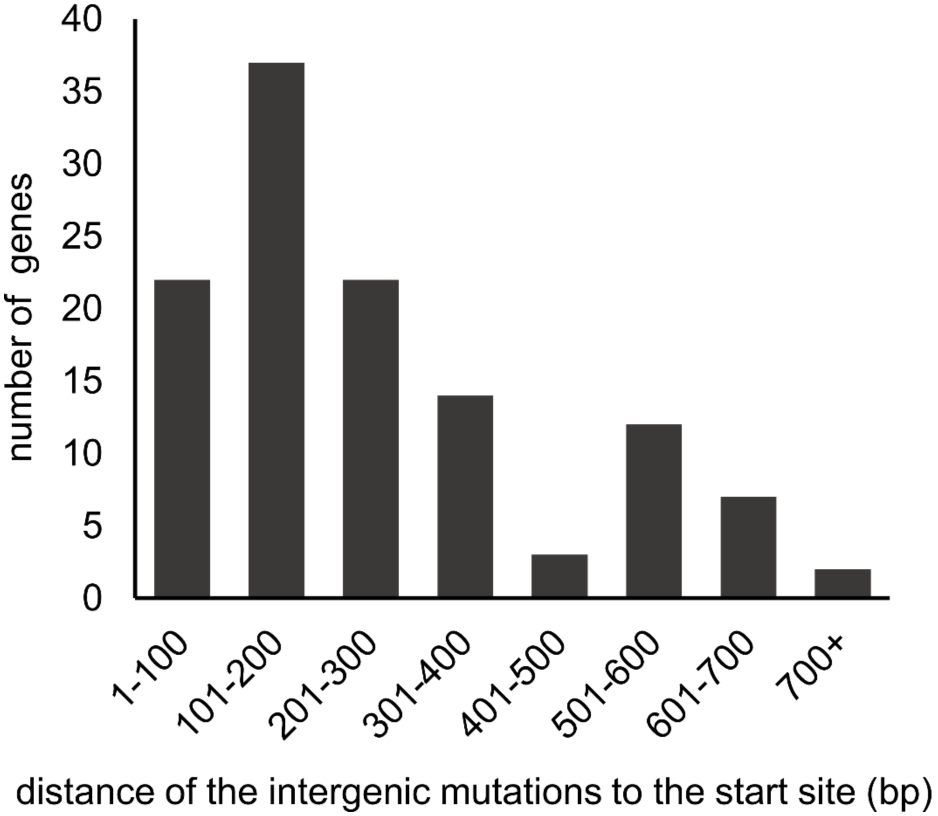

Supplementary Figure 11. Distance of intergenic mutations to the start site of proximal genes. Mutations were grouped into 100 bp windows shown on X axis, and Y axis shows the number of genes in each group.

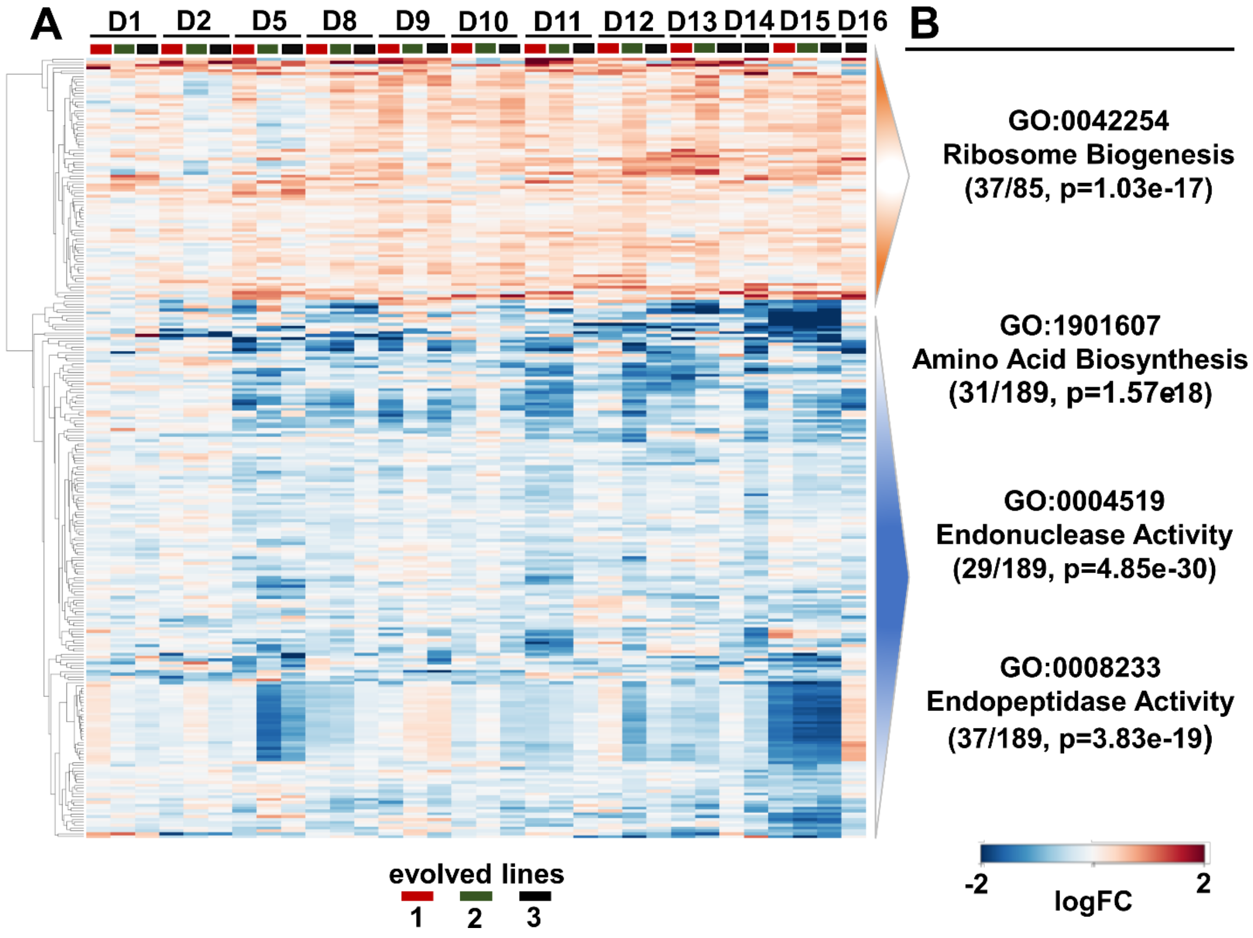

Supplementary Figure 12. Functional enrichment of genes co-regulated across evolved lines. **(A)** Heatmap representation of genes that commonly changed expression across evolved lines of disomic strains. Red represents 85 up-regulated genes and blue 189 down-regulated genes across evolved lines. Evolved lines of each disomic strains are shown in green (evolved line 1), red (evolved line 2) and black (evolved line 3). **(B)** Enriched GO terms for genes with increased and decreased expression across evolved lines, analyzed with gprofiler tool. The number of genes per each category is indicated in parenthesis, wherein the first number corresponds to the total number of upregulated or down regulated genes and the second number indicates the number of genes in this GO category. P-values for each GO category are also shown in parentheses.

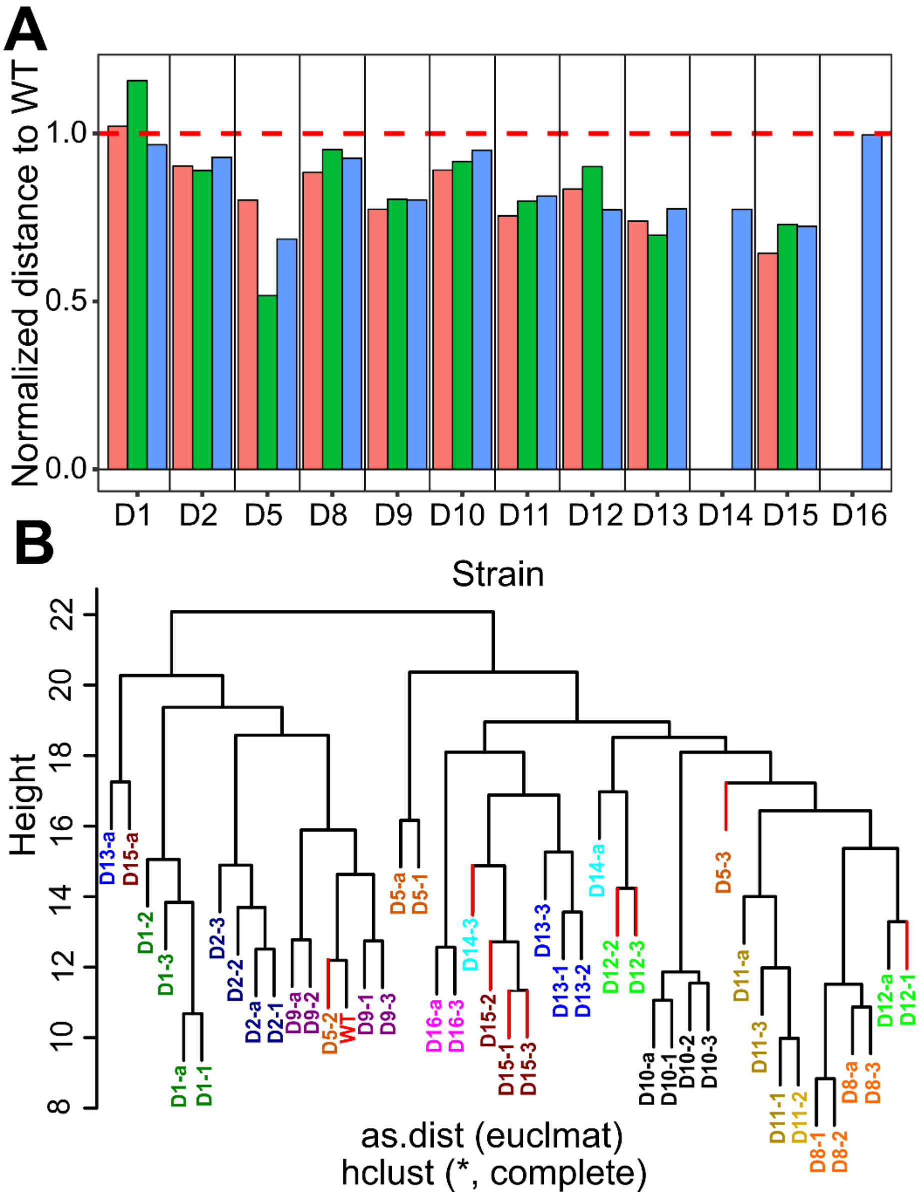

Supplementary Figure 13. Expression dynamics of evolved disomic strains. (A) Plot shows gene expression distance between evolved samples and WT (as defined in Methods). Note that this distance metrics considers only those genes that were altered in the ancestor disomic lines. The great majority of lines shifted their expression towards WT during adaptive evolution. (B) Hierarchical clustering of RNAseq samples used in our study based on the Euclidean distances of genome-wide transcript levels in log space. Note that, unlike in panel A, this distance considers all genes. The tree was constructed by complete hierarchical clustering. Generally, evolved strains clustered together with their ancestor strains. Red branches indicate strains with total or partial loss of duplicated chromosomes.

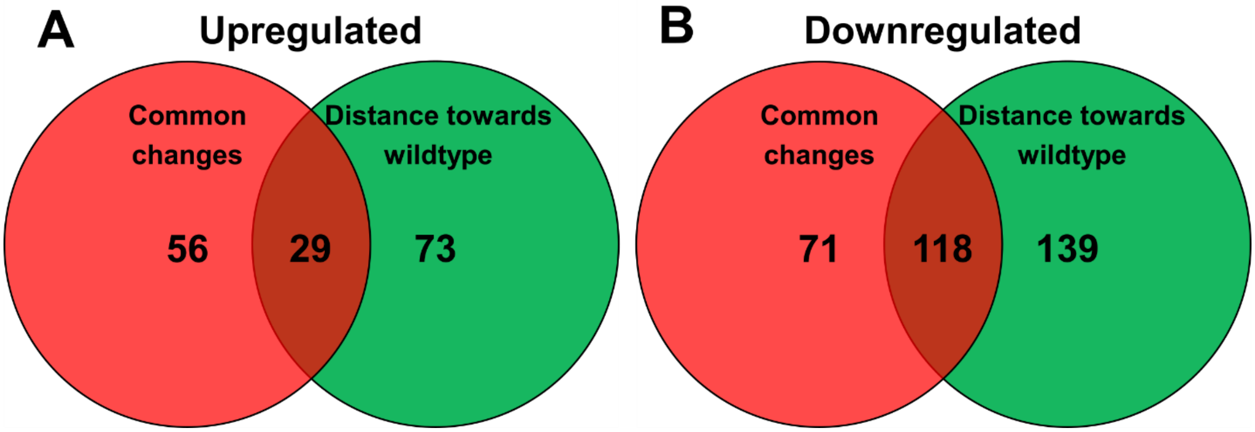

Supplementary Figure 14. Overlap of gene expression shift-associated genes and commonly regulated genes. Venn diagram shows overlap between genes commonly changed across all the evolved disomic lines and genes associated with the distance towards WT across all evolved lines. **(A)** Upregulated genes. **(B)** Down-regulated genes.

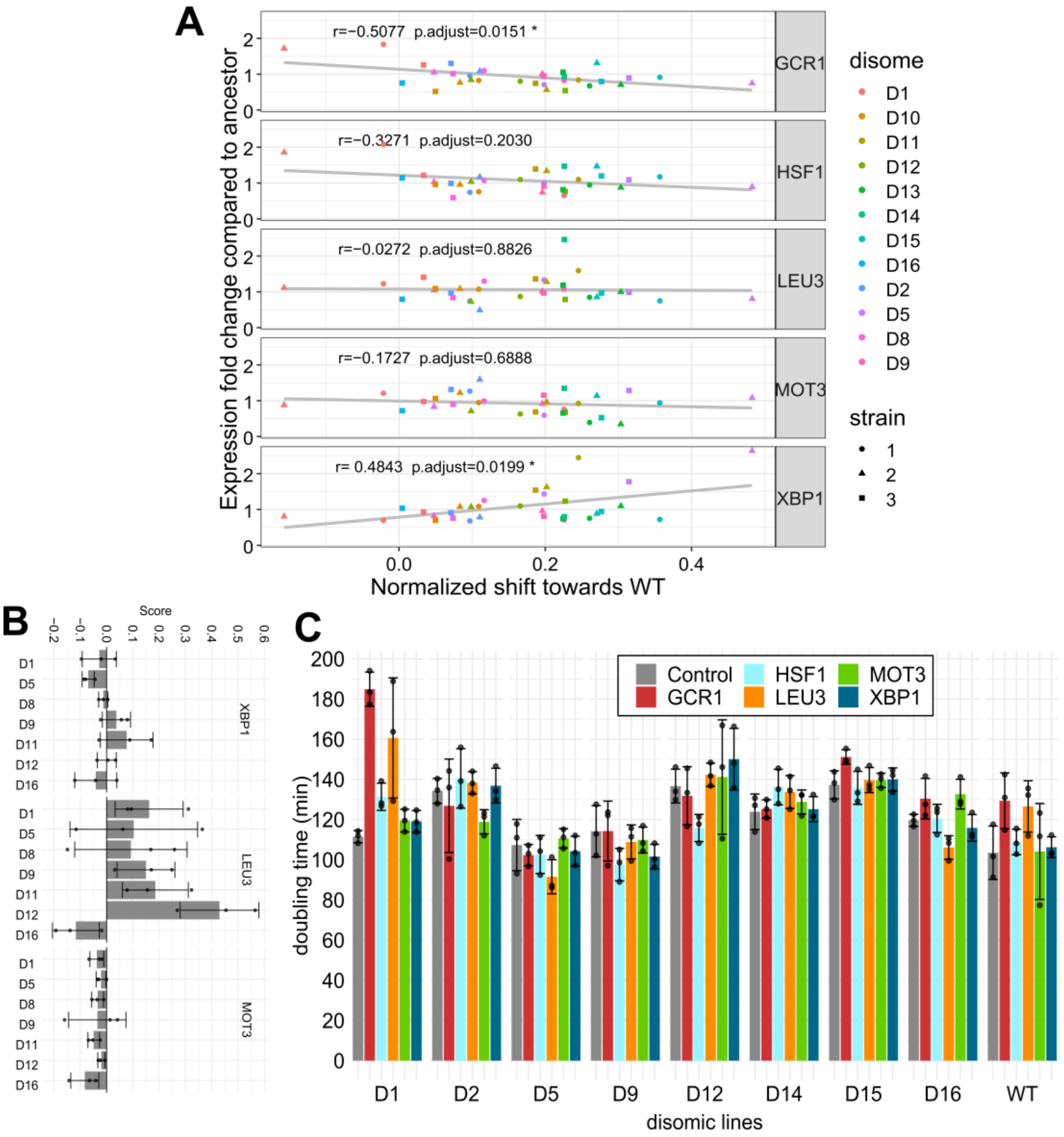

Supplementary Figure 15. Characterization of the effect of transcription factors. (A) RNA expression changes of five TFs in evolved disomic lines. For two TFs (GCR1 and XBP1), expression fold-change between evolved and ancestral disomes correlated significantly with the normalized expression shift towards WT. **(B)** The effect of deletion of 3 non-essential TFs. GCR1 and HSF1 could not be analyzed as these two TFs are essential. **(C)** Overexpression of five TFs analyzed across 8 disomic and WT strains. Gray bars indicate control cells that harbor an empty vector, and cells expressing one of TFs are shown with different colors: GCR1-red, HSF1-blue, LEU3-orange, MOT3-green and XBP1-dark blue. Error bars show standard error of 3 replicates and significance of growth difference between each strain is included in Supplementary Data 6. Source data for panel A and 5 is provided as source data file and source data of doubling times for each strains is provided as Supplementary Data 6.

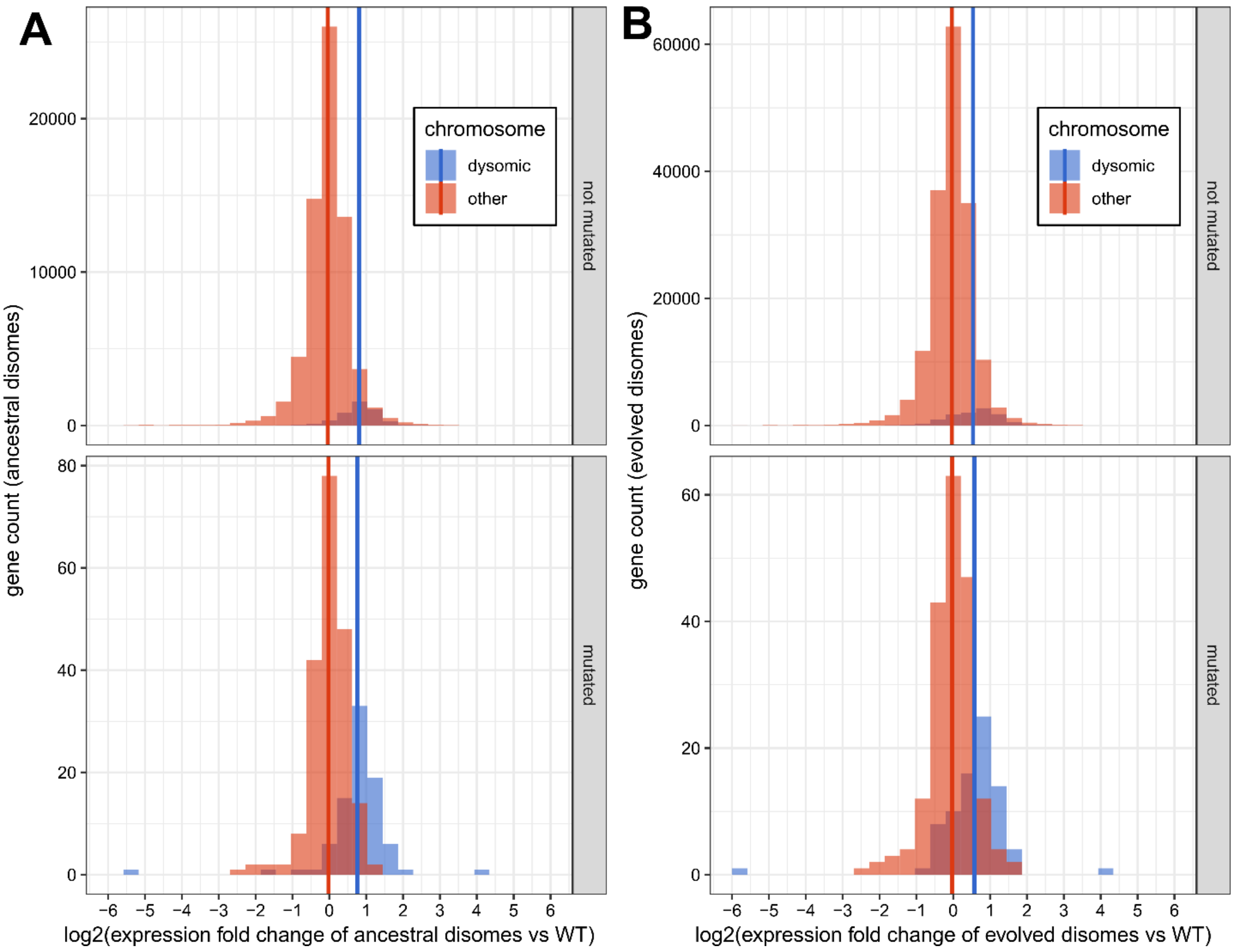

Supplementary Figure 16. Expression profiles of genes with and without mutations in evolved disomic lines. The plot shows fold change in gene expression compared to WT of (A) ancestral disomic lines, and (B) evolved disomic lines. Colors are used to distinguish between the genes on the disomic chromosome (e.g., those on chromosome XI for D11; blue) and the others (red). Vertical lines show the average values per group. In panel A, every gene is considered once for each different disome, while in panel B every gene is considered once for each disomic line. On top, the plot shows those genes that accumulated at least one mutation in any evolved line for a given disome (panel A), or at least one mutation in a given evolved line (panel B). The average expression change of genes on disomic chromosomes is nearly the same for mutated and non-mutated group, both at the beginning (A) or at the end (B) of the evolution experiment, whether we consider fold change to WT or the log2 of fold change (two-sided t-test p-value >0.6 in all cases).

REFERENCES

1. Zhu, Y.O., Sherlock, G. & Petrov, D.A. Whole genome analysis of 132 clinical Saccharomyces cerevisiae strains reveals extensive ploidy variation. G3 (Bethesda) 6, 2421-31 (2016).
2. Peter, J. et al. Genome evolution across 1,011 Saccharomyces cerevisiae isolates. Nature 556, 339-44 (2018).
