# Supplementary material for: Molecular signatures of aneuploidy-driven adaptive evolution
Source: Nat Commun. 2020 Jan 30;11:588. doi: 10.1038/s41467-019-13669-2 (PMC6992709; doi:10.1038/s41467-019-13669-2)
Supplement: Supplementary file 3 — Description of Additional Supplementary Files [file 41467_2019_13669_MOESM3_ESM.pdf]

**Description of Additional Supplementary Files**

File Name: Supplementary Data 1
Description: List of mutations identified in this study.

File Name: Supplementary Data 2
Description: Commonly mutated genes and genes with mutations in the promoter region in more than one evolved disomic line.

File Name: Supplementary Data 3
Description: Raw values of doubling times in disomic strains expressing SCH9 mutated alleles together with p-values.

File Name: Supplementary Data 4
Description: Raw read counts of RNAseq data.

File Name: Supplementary Data 5
Description: List of genes correlating with expression shift towards WT and commonly regulated genes across evolved lines.

File Name: Supplementary Data 6
Description: Raw values of doubling times in disomic strains expressing different TFs together with p-values.
